# Multi-Omics Characterization of Quality Attributes in Pigeon Meat

**DOI:** 10.3390/foods14183230

**Published:** 2025-09-17

**Authors:** Xinran Wang, Yunyun Hu, Yan Liu, Cheng Li, Zheng Wang, Meiyu Liu, Jinhui Zhou, Meng Wang

**Affiliations:** 1 Institute of Quality Standard and Testing Technology, Beijing Academy of Agriculture and Forestry Sciences, Beijing 100097, China; xinran_w@126.com (X.W.); lic@iqstt.cn (C.L.); 2 Institute of Food Science and Technology, Chinese Academy of Agricultural Sciences, Beijing 100193, China; 17856385322@163.com (Y.H.); 821012450630@caas.cn (Y.L.); 3 Institute of Animal Husbandry and Veterinary Medicine, Beijing Academy of Agriculture and Forestry Sciences, Beijing 100097, China; wz7324@163.net; 4 School of Life Sciences and Food Engineering, Hebei University of Engineering, Handan 056000, China; lmy200751@163.com

**Keywords:** pigeon meat, chicken meat, quality, multi-omics technology, lipids

## Abstract

Pigeon meat is gaining increasing popularity due to its high nutritional value and desirable sensory qualities. This study aimed to comprehensively evaluate the quality-related components of pigeon meat by analyzing conventional nutritional indicators—including amino acids, fatty acids, and flavor nucleotides—in combination with multi-omics approaches. The results indicated that pigeon meat contains high levels of arginine (Arg), alanine (Ala), linoleic acid, and glycerophospholipids (GPs), which contribute significantly to its flavor profile. Additionally, several lipids, namely, PS (18:0/20:4), PE (16:2; O/2:0), HexCer (9:0;2O/42:11), Hex2Cer (38:1;2O), PS (16:0; O/21:0), and PE (42:9), were identified as potential characteristic markers of pigeon meat. A comparative analysis among three breeds—White King, Shiqi, and Tarim pigeons—revealed breed-specific differences in endogenous compounds, with each breed exhibiting distinct compositional traits. This study provides a comprehensive dataset for quality assessment and offers critical insights for the authenticity verification of pigeon meat.

## 1. Introduction

With the rise in living standards, consumers are increasingly demanding higher-quality meat products. Pigeon meat, known for being low in fat and high in protein, accounts for a substantial portion of meat consumption in Asia, particularly in China [1]. According to reports, China has maintained approximately 45 million pairs of breeding pigeons, with around 740 million squabs sold over the past five years [2]. Furthermore, pigeons have become the fourth major category of poultry in China, contributing to over 80% of global production. Currently, China’s pigeon meat industry relies mainly on imported breeds, among which the White King pigeon (WK) represents the largest share of the domestic stock. Additionally, several indigenous breeds, such as the Shiqi pigeon (SQ) and Tarim pigeon (TR), also serve as important meat pigeon varieties within the country and hold a considerable market share [3]. Rich in amino acids, trace elements, and chondroitin, pigeon meat is suggested to offer various physiological benefits, including promoting bone development, preventing osteoporosis, improving human nutrition, aiding wound healing, and reducing hair loss [4,5,6]. Therefore, the analysis of pigeon meat quality characteristics is essential for exploring its utilization and developmental value, and holds significant importance for the advancement of the pigeon industry.

However, compared to other poultry such as chicken and duck, studies on the nutritional composition of pigeons remain relatively limited. To date, research on pigeon meat quality has primarily focused on basic parameters, including color, pH, water loss rate, shear force, and conventional nutritional components. Only a few studies have reported the contents of compounds such as inosine monophosphate (IMP), total amino acids, and fatty acids in pigeons [3,7,8]. Some researchers have also explored the effects of nutritional supplements, such as vitamin B2, on pigeon meat quality, including fatty acid profiles and antioxidant activity [6]. To our knowledge, no comprehensive analysis of quality components in pigeon meat using multi-omics technologies has been reported, which hinders the full exploitation and consumer understanding of pigeon meat quality.

In recent years, multi-omics technologies, including metabolomics, lipidomics, and proteomics, have been widely applied in meat quality evaluation [9]. Metabolomics enables the identification and quantification of small molecules in biological systems, such as free amino acids, short peptides, monosaccharides, organic acids, lipids, vitamins, and nucleotides. It provides a comprehensive profile of metabolites in a given sample, offering an effective approach for analyzing the chemical composition of poultry meat and identifying diverse metabolites. Recent applications of metabolomics have included characterizing breed-specific metabolic profiles in meat [10,11], as well as elucidating the formation mechanisms of quality-related components during livestock and poultry growth [12,13,14]. As a subfield of metabolomics, lipidomics focuses on the identification, quantification, and spatial distribution of lipids, facilitating the analysis of their biological roles within metabolic pathways and networks. It has proven to be an efficient method for comprehensive lipid profiling in meat [15]. Previous studies have successfully revealed lipid characteristics in poultry meats such as chicken, duck, and goose across different breeds, cuts, and feeding regimens [10,16]. Moreover, integrated metabolomic and lipidomic approaches, combined with volatile compound analysis, have helped identify key flavor precursors in processed meat products. For instance, compounds such as oxoglutaric acid, fumarate, and L-aspartate have been identified as important flavor contributors in Laiwu pork and Nuodeng ham [17,18], while phosphatidylcholine (PC) and phosphatidylethanolamine (PE) were shown to play key roles in aroma formation in roasted mutton [19]. Therefore, the application of multi-omics technologies for the comprehensive quality analysis of poultry meat is also highly relevant for future research on flavor formation in processed pigeon products.

The objective of this study was to characterize the composition and content of quality-related components in pigeon meat using multi-omics technology. Conventional nutritional parameters, including amino acids, fatty acids, and flavor nucleotides, were also determined and compared with those of chicken. Furthermore, quality differences among three pigeon breeds (WK, SQ, and TR) were evaluated based on these indicators. This study presents the first comprehensive analysis of flavor-related substances in pigeon meat, providing accurate data for quality assessment and a theoretical foundation for the utilization and development of pigeon meat resources.

## 2. Materials and Methods

### 2.1. Animals and Sample Collection

All animal welfare practices and experimental procedures were performed in accordance with the Guide for the Care and Use of Laboratory Animals (Ministry of Science and Technology of China, 2006 [20]). All procedures were approved by the Animal Ethics Committee of Beijing Academy of Agriculture and Forestry Sciences (IHVM11-2312-53).

A total of 30 pigeons aged 28 days, 10 each of WK, SQ, and TR pigeons, were collected from a local experimental base (Beijing, China) with similar feeding management systems and diets (cereal and silage). Thirty chicken samples were used as control samples, including Taihang, Beijing You, and Jingfen chickens. The diet was cut off the night before slaughter (12 h fast). The animals were transported to a corresponding slaughterhouse and slaughtered in accordance with standard procedures [International Code of Practice for Slaughtering Animals (CAC/RCP 41-1993) [21] and Animal Welfare Management (ISO/TS 34700: 2016)] [22]. After slaughter, the tendons and connective tissue were cleaned and removed from the breast muscles. The remaining breast muscles were immediately frozen and stored at −20 °C until analysis.

### 2.2. Free Amino Acid Analysis

The determination of free amino acid was performed using the previous method [23], 5 g of poultry meat was placed into a 50 mL polypropylene centrifuge tube, and then 15 mL of 0.02 mol/L hydrochloric acid solution was added and vortexed until the sample had completely dissolved. The mixture was brought up to 25 mL, and then centrifuged for 10 min at 8800 rpm and at 4 °C. Then, 2 mL of supernatant was taken out, and 2 mL of 8% salicylic acid solution was added. After being kept at 4 °C for 30 min and centrifuged for 10 min at 10,000 rpm, the supernatant was removed and cleaned with 2 mL n-hexane. The mixture was centrifuged for 10 min at 10,000 rpm before being filtered through a 0.22 μm membrane prior to analysis with an L-8900 amino acid automatic analyzer (Hitachi High-tech Co., Ltd., Shanghai, China).

### 2.3. Flavored Nucleotide Analysis

The determination of flavored nucleotides in the poultry meat was performed according to a previously described method [23]. Briefly, 2 g of poultry meat was placed into a 50 mL polypropylene centrifuge tube, and then 10 mL of 5% perchloric acid solution as an extraction solution was added to the samples. The mixture was vortexed and ultrasonicated for 10 min until homogeneous mixing was achieved. The samples were centrifuged for 10 min at 8800 rpm at 4 °C. Then, the upper layer was transferred into another polypropylene centrifuge tube, 10 mL of the extraction solution was added to the residual precipitate, and the extraction procedure was repeated. The upper layer was combined and adjusted to pH 6.5 with 5% NaOH. After filling to 30 mL, 3 mL of the extraction solution was loaded onto a 3 mL Captiva EMR-lipid cartridge. The eluent was collected and vortexed, and 0.1 mL of eluent was diluted with 0.9 mL of a 0.1% formic acid solution and fully mixed. Finally, the 1 mL reconstitution solution was passed through a 0.22 μm filter membrane before injection.

The analysis of nucleotides was performed using an 8060NX high-performance liquid chromatograph (HPLC) (Shimadzu, Kyoto, Japan) coupled with an 8060 triple quadrupole tandem mass spectrometer (Shimadzu, Kyoto, Japan). Chromatographic separation was carried out using an ACQUITY UPLC BEH C18 column (Waters, Milford, MA, USA) (2.1 × 100 mm, 1.7 μm) at 35 °C. The mobile phase contained 0.2% formic acid in 5 mM ammonium acetic and 0.2% formic acid in acetonitrile. The linear gradient elution was as follows (in% B): 0 min, 1%; 0.8 min, 1%; 2.5 min, 8%; 4 min, 20%; 6 min, 90%; 7 min, 90%; 7.1 min, 1%. The flow of the mobile phase was set at 0.35 mL/min with an injection volume of 2 μL. The analysis in the MS/MS system was conducted via multiple reaction monitoring (MRM) with the ESI source in positive ionization mode. The following instrument conditions were used: atomizing gas flow, 3 L/min; interface temperature, 400 °C; desolvation temperature, 250 °C; desolvation gas flow, 15 L/min; drying gas flow, 3 L/min. The MRM parameters for the seven target analytes are summarized in Table S1.

### 2.4. Fatty Acid Analysis

The determination of the fatty acid in the poultry meat was performed according to a previously described method with minor modifications [24]. Here, 1 g of the meat sample was weighed into a centrifuge tube. After adding 8 mL of chloroform/methanol (2:1, *v*/*v*) as the extraction solution, the sample was vortexed for 60 s and sonicated for 60 min at 60 °C. The chloroform layer was taken out, 8 mL 5% sulfuric acid in methanol was added, and the sample was kept at 80 °C for 0.5 h. Then, 2 mL of n-hexane was added for extraction, and 5 mL of water was used for washing. The supernatant was taken out, and 2 mL of n-hexane was added to the remaining solution for extraction. Finally, the supernatant was combined and filtered through a 0.22 μm nylon membrane prior to the gas chromatography–mass spectrometry (GC-MS) analysis.

The GC-MS analysis was performed using a QP2010Plus GC-MS instrument (Shimadzu, Japan) equipped with a Supelco SPTM-2560 capillary column (Supelco, Bellefonte, PA, USA) (100 mm × 0.25 mm × 0.2 μm). The injector was used in split mode with a split ratio of 50:1. The column flow rate was 1 mL/min with high-purity helium (>99.999%) as the carrier gas. The GC oven temperature was maintained at 140 °C for 4 min after sample injection and then increased by 4 °C/min to 240 °C and held for 30 min. The injector temperature was set at 200 °C. The temperature of the transfer line was 240 °C. The temperature of the ion source was set to 230 °C. The mass spectrometer was operated in electron-impact mode at 70 eV, and full scan mass spectra were collected in the range of 33–1500 *m*/*z*.

### 2.5. Metabolomics Analysis

Briefly, 500 mg samples were extracted using methanol/acetonitrile/water (2:2:1, *v*/*v*/*v*) and vortexed for 5 min. The samples were processed in an ice ultrasonic crusher for 30 min, kept at −20 °C for 30 min, and then centrifuged for 15 min (13,000 rpm, 4 °C). The supernatant was combined and filtered through a 0.22 μm nylon membrane prior to the LC-Q-TOF analysis. The quality control (QC) samples were prepared by mixing all samples of meat from both pigeons and chickens to evaluate the repeatability and robustness of the analyses by interspersing QC determinations throughout the whole sequence.

The LC-Q-TOF analysis was performed using a lipid chromatography system coupled with time-of-flight mass spectrometry (Waters Xevo G2 QTOF, Waters, Milford, MA, USA). LC separations were performed on a Waters ACQUITY UPLC HSS T3 column (2.1 × 100 mm, 1.7 µm) with a flow rate of 0.3 mL/min at 30 °C. The mobile phase consisted of 20 mmol/L ammonium acetate in water (solvent A) and 0.1% formic acid in acetonitrile (solvent B). The samples were eluted with the following linear gradient: 0–2 min, 5–30% B; 2–21 min, 30–95% B; 21–24 min, 95% B; 24.5–29 min, 5% B. The injection volume was 5 µL. MS data were acquired in MSE mode under sensitivity mode in both positive and negative electrospray ionization (ESI+ and ESI-). The acquisition range was 50–1000 *m*/*z*. The capillary voltage was 3.2 kV (ESI+) and 3.0 kV (ESI-), and the cone voltage was 30 V for both. The source offset was 80 V, and the cone gas flow was set at 50 L/h with a source temperature of 100 °C. The desolvation gas flow was maintained at 350 L/h with a desolvation temperature of 350 °C. The extraction cone voltage was set to 2 V, ion energy to 1 V, and collision energy to 10 V. The data were processed using MassLynx V4.1 and Progenesis QI V2.0 software.

### 2.6. Lipidomics Analysis

For this analysis, 20 mg samples of breast meat were weighed into a 2 mL centrifuge tube, and 200 μL of water and 480 μL of MTBE-MeOH (5:1, *v*/*v*) were added as the extraction solution. Then, the samples were vortexed for 60 s, homogenized at 35 Hz for 4 min, and sonicated for 5 min in an ice water bath. The homogenate and sonicate steps were repeated three times. The extraction solution was kept at −40 °C for 1 h, and the samples were centrifuged at 3000 rpm for 15 min at 4 °C. Then, 300 μL of the supernatant was dried in a low-temperature vacuum. The dried samples were reconstituted in 200 μL of DCM-MeOH-H_2_O (6:3:1, *v*/*v*/*v*), vortexed for 60 s, and sonicated for 5 min in an ice water bath. The sample was then centrifuged at 12,000 rpm for 15 min at 4 °C, and the supernatant was transferred to a 2 mL vial until analysis. The QC samples were prepared in the same manner as those for the metabolomics analysis.

A UPLC/Orbitrap MS system consisting of a Thermo Scientific Ultimate 3000 system and an Orbitrap Exploris 120 mass spectrometer (Thermo Fisher Scientific, Waltham, MA, USA) was used to screen and quantitatively analyze the lipid compounds. The UPLC/Orbitrap MS system control was performed by Xcalibur 4.4. Liquid chromatography was carried out using a Phenomenex Kinetex C18 (Phenomenex, Los Angeles, CA, USA) (2.1 mm × 100 mm, 2.6 μm) with a flow rate of 0.3 mL/min at 40 °C. The mobile phase consisted of H_2_O–acetonitrile (6:4, *v*/*v*) with 10 mM ammonium formate as solvent A and isopropanol–acetonitrile (9:1, *v*/*v*) with 10 mM ammonium formate as solvent B. The samples were eluted with a linear gradient from 37% B to 98% B over 20 min, followed by 98% B for 6 min and 4 min re-equilibration with 37% B. The injection volume was 2 μL. The ESI source conditions were set as follows: sheath gas flow rate, 30 Arb; Aux gas flow rate, 10 Arb; capillary temperature, 320 °C; full MS resolution, 60,000; MS/MS resolution, 15,000; collision energy, 15/30/45 eV; and spray voltage, 3.8 kV (positive) or −3.4 kV (negative), respectively.

### 2.7. Statistical Analysis

In this study, three replicates were performed for each sample. In order to highlight the quality characteristics of pigeon meat, the comparison samples of chicken meat were regarded as a whole in the data analysis. The data were expressed as mean ± standard deviation. Then, a one-way analysis of variance (ANOVA) followed by Tukey’s multiple comparisons test was performed to assess the significance of the samples (*p* < 0.05) using SPSS 22.0 software (SPSS Inc., Chicago, IL, USA). Origin 2021 software was used for data visualization. SIMCA-P software (Version 14.1, Umetrics AB, Umeå, Sweden) and “MetaboAnalyst” (https://www.metaboanalyst.ca/) were simultaneously applied to analyze the omics data, build the discriminant analysis model (PCA and OPLS-DA), and search for characteristic quality components. For the omics data, variance homogeneity was assessed internally via Levene’s test, with Welch’s correction applied where appropriate. Non-normally distributed data were analyzed using the Mann–Whitney U test.

## 3. Results and Discussion

### 3.1. Free Amino Acid

The amino acid compositions of pigeon and chicken meat are presented in Table 1. A total of 16 amino acids were detected in both types of meat, with glutamate (Glu), alanine (Ala), aspartate (Asp), and arginine (Arg) exhibiting the highest concentrations. Notably, the proportion of essential amino acids in pigeon meat (approximately 39.17%) was significantly higher than that in chicken, indicating that pigeon meat may provide higher-quality protein for human consumption. Phenylalanine (Phe), leucine (Leu), isoleucine (Ile), and valine (Val) were present at higher levels in pigeon meat and can be regarded as characteristic amino acids of this protein source. Amino acids play a crucial role as flavor components in poultry meat. Free amino acids can participate in Maillard reactions with reducing sugars to generate various heterocyclic compounds—such as furans, pyrazines, pyrroles, oxazoles, thiophenes, and thiazoles—that contribute significantly to the characteristic flavor of meat products [25]. For instance, hydrogen sulfide derived from the Strecker degradation of cysteine serves as a precursor for 2-furfuryl mercaptan, an important aroma compound in roasted meat [26]. In this study, the proportion of umami amino acids was significantly higher (*p* < 0.05) in pigeon meat than in chicken, suggesting a more intense flavor profile. Specifically, the contents of Arg and Ala were significantly elevated in pigeon meat and can be considered characteristic of its flavor. Ala, a sweet-tasting amino acid, not only imparts sweetness, but also mitigates bitterness in meat [27]. Previous studies have indicated that bitter amino acids such as Arg, Phe, and tyrosine (Tyr)—when present below their taste thresholds—can enhance the umami and sweet notes of other amino acids [27]. In pigeon meat, the content of these bitter amino acids, particularly Arg, remained below the taste threshold, thereby contributing to taste complexity and enhancing overall savoriness. Moreover, methionine (Met) was found at higher levels in pigeon meat and may also serve as a characteristic amino acid. Although Met does not directly contribute to flavor, it participates in Maillard reactions during thermal processing, thereby influencing the flavor profile of pigeon meat products. Collectively, Arg, Ala, and Met synergistically contribute to the distinctive and desirable taste of pigeon meat.

Subsequently, variations in free amino acid profiles among different pigeon breeds were analyzed. The White King (WK) breed exhibited the highest total free amino acid content (194.88 mg/100 g), followed by Shiqi (SQ, 150.00 mg/100 g) and Tarim (TR, 137.35 mg/100 g). The WK breed also showed the highest content and proportion of essential amino acids, consistent with findings reported by Li [3]. However, SQ and TR pigeons contained a higher proportion of umami amino acids. Glu, the most abundant free amino acid in all pigeon breeds, showed significant differences among breeds, with TR containing significantly lower levels than both WK and SQ.

### 3.2. Flavored Nucleotides

Nucleotides represent another important source of umami flavor in poultry meat. As shown in Table 1, seven types of flavor nucleotides were detected in both pigeon and chicken meat. Among these, IMP—derived from the degradation of adenosine triphosphate—was the most significant flavor nucleotide, with a Taste Activity Value (TAV) exceeding 1, indicating its contribution to the taste profile of both types of meat. IMP is also known to synergize with guanosine monophosphate (GMP) to enhance umami perception [28]. In addition to IMP, inosine (I) was also found at relatively high levels in both pigeon and chicken meat.

Compared to the difference between pigeon and chicken meat, the total content of flavor nucleotides varied more significantly (*p* < 0.05) among different pigeon breeds. The total amount of flavor nucleotides was considerably higher in SQ and TR than in WK, primarily due to the higher IMP content in the former two breeds. In contrast, WK exhibited a higher inosine content, which accounted for 38.91% of its total flavor nucleotides.

### 3.3. Fatty Acid

A total of 27 fatty acids were identified in pigeon and chicken meat, comprising 10 saturated fatty acids (SFAs), 7 monounsaturated fatty acids (MUFAs), and 10 polyunsaturated fatty acids (PUFAs). Among these, myristic acid and arachidonic acid were detected exclusively in pigeon meat.

In terms of content, the average concentration of fatty acids in pigeon meat (2.39 g/100 g) was significantly higher than that in chicken meat (0.90 g/100 g). Unsaturated fatty acids, particularly PUFAs, play important physiological roles in humans, such as improving cardiovascular health and supporting cellular function and metabolism. Although the proportion of unsaturated fatty acids was lower in pigeon meat than in chicken, the total amount of unsaturated fatty acids—especially ω-6 PUFAs—was significantly higher in pigeon meat.

ω-6 PUFAs are essential fatty acids for animals and are closely associated with lipid metabolism, immune function, and reproductive performance. They contribute to the regulation of animal health and product quality. Among all ω-6 PUFAs, linoleic acid was the most abundant in pigeon meat, with a content exceeding 0.17 g/100 g. Linoleic acid is also recognized as an important flavor precursor in poultry. During heating, it undergoes oxidation catalyzed by lipoxygenases, transition metal ions, or free radicals, forming primary oxidation products. These compounds further degrade or recombine to generate volatile flavor substances such as aldehydes, alcohols, ketones, and furans. Additionally, oxidation products of linoleic acid can interact with Maillard reaction intermediates to form aldehydes and other compounds that contribute roasted and other desirable aromas to poultry meat [29,30]. Thus, linoleic acid serves as a characteristic fatty acid in pigeon meat, enhancing its quality and providing flavor precursors for processed pigeon products.

**Figure 1:**
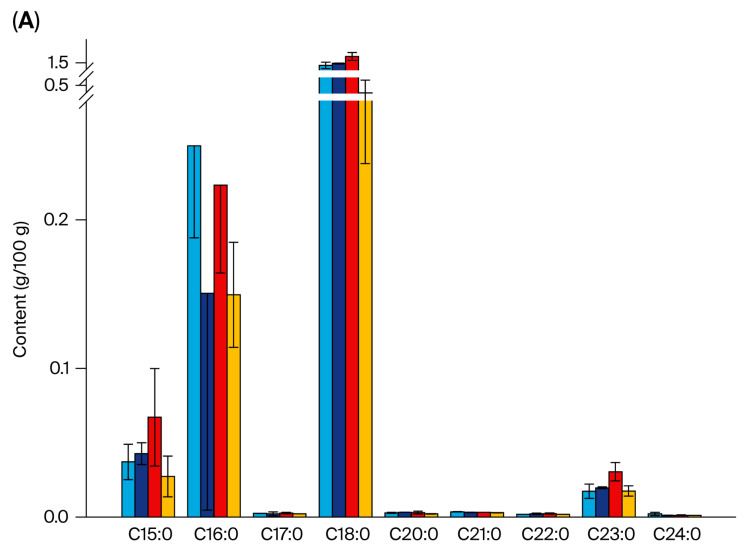

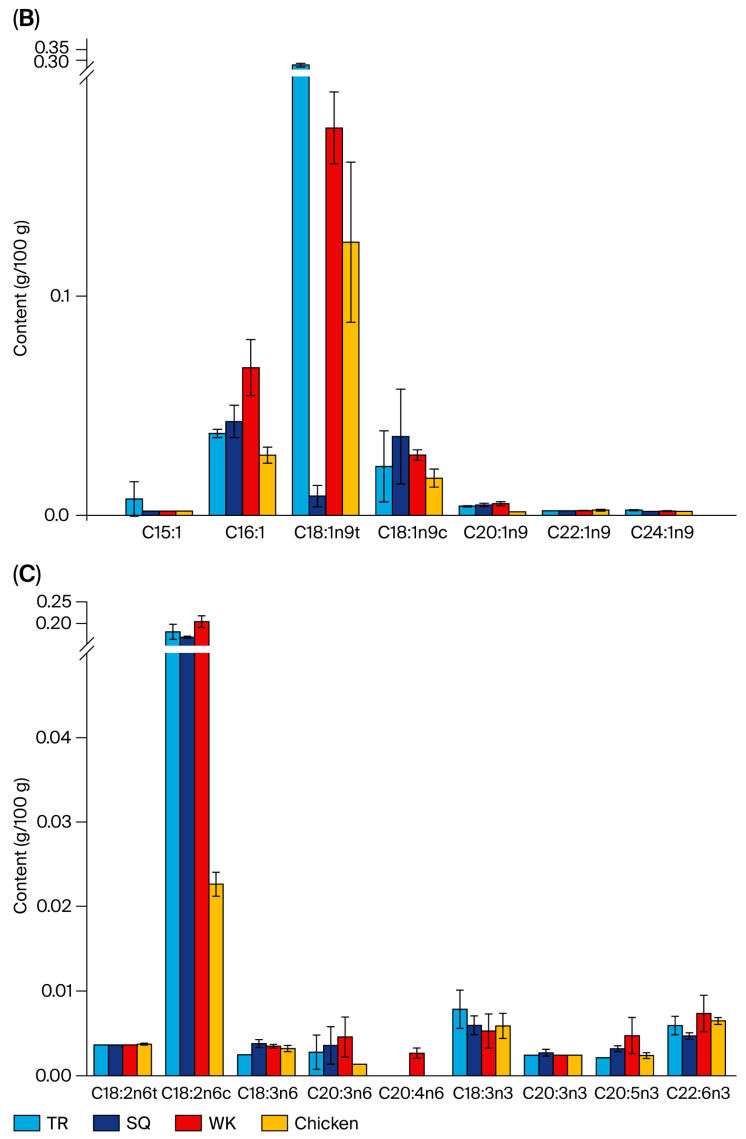
The fatty acid content in chicken and three breeds of pigeon. (**A**) The contents of SFAs; (**B**) The contents of MUFAs; (**C**) The contents of PUFAs.

The analysis of fatty acids across the different pigeon breeds revealed significant variations in total free fatty acid content. The highest level was observed in WK, followed by TR, while SQ showed the lowest. Myristic acid was only detected in TR, and arachidonic acid exclusively in WK, indicating that these can be considered breed-specific characteristic fatty acids. As illustrated in Figure 1, the proportion of unsaturated fatty acids in TR (31.98%) was substantially higher than in the other two breeds. Moreover, the proportion of ω-6 PUFAs in SQ and TR exceeded 9%, which was significantly higher than that in WK. In terms of the content of individual fatty acids, palmitoleic acid and elaidic acid showed significant differences in the meat from the different breeds of pigeon.

### 3.4. Metabolomics Analysis

**Figure 2:**
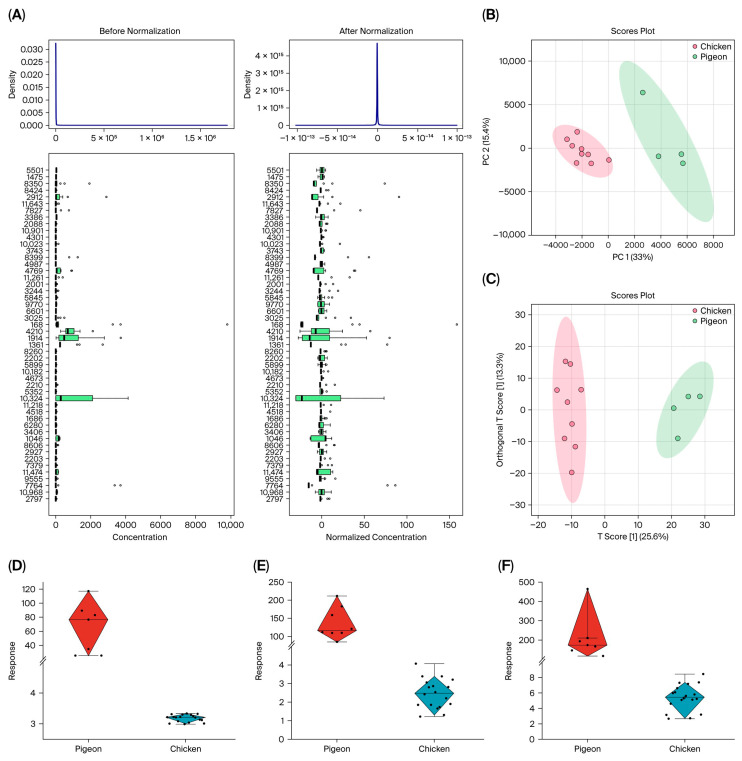
Differential analysis of pigeon meat and chicken based on metabolomics. (**A**) The impact of data preprocessing on the distribution of data; (**B**) PCA score plot of pigeon and chicken meat; (**C**) OPLS-DA score plot of pigeon and chicken meat; (**D**) content of palmitic amide in pigeon and chicken; (**E**) content of tetrahydrocortisone in pigeon and chicken; (**F**) content of mesaconic acid in pigeon and chicken.

To characterize the quality attributes of pigeon meat, non-targeted metabolomics analysis was conducted using LC-Q-TOF technology. The results revealed a total of 11,648 metabolites identified across both negative and positive ion modes. The raw metabolomics data were preprocessed using Pareto scaling to mitigate model errors arising from differences in variable magnitudes, thereby transforming the data into concentration differences relative to the scaling factor applied in this study (Figure 2A). Following normalization, the data approximated a normal distribution.

To elucidate the metabolic characteristics of pigeon meat, non-targeted metabolomics was performed using LC-Q-TOF technology. Initially, an unsupervised PCA model was employed to assess the overall metabolic differences between pigeon and chicken. As shown in Figure 2B, a clear separation was observed between the two species in the PCA score plot. The combined contribution rate of PC1 and PC2 was 48.4%, with individual contributions of 33% and 15.4%, respectively. The 95% confidence ellipses confirmed distinct clustering in each group. All samples fell within Hotelling’s T^2^ limit (T^2^crit (95%) = 7.84), and no outliers were detected.

Subsequently, a supervised OPLS-DA model was applied to further extract characteristic metabolites and identify the quality-related features of pigeon meat (Figure 2C). The model demonstrated high goodness-of-fit and predictive ability, with R^2^Y = 0.86 and Q^2^ = 0.83. A permutation test (200 iterations) confirmed that the model was robust and not overfitted. Differential metabolites were screened based on VIP ≥ 1.5, *p* < 0.05, and fold change ≥20 or ≤0.05, yielding 472 potential differential ion peaks. These ions were annotated using public databases, including HMDB, Metlin, *mz*Cloud, and KEGG, with a mass tolerance of ±5 ppm, and further verified by comparing retention times and mass fragments with reference standards. Metabolites with high error rates (MS/MS spectral match scores < 70%) or inconsistent retention times were excluded from the analysis.

A total of 105 metabolites were identified as significantly more abundant in pigeon meat. These were primarily categorized as amino acids/peptides (14), lipids (12), organic acids (8), nucleotides (4), and others. Notably, lipids and nucleotides—such as glycerophospholipids, IMP, and inosine—were markedly higher in pigeon than in chicken. IMP, a key flavor nucleotide, is unstable in muscle and can degrade into inosine [31]. Additionally, small molecules such as homovanillic acid, glycitin, and tauroursodeoxycholic acid exhibited significant differences and may serve as characteristic markers of pigeon meat, with response values exceeding those in chicken by more than 50-fold (Figure 2D–F).

Furthermore, the same metabolomics approach was applied to compare different pigeon breeds. A total of 23 differential compounds were identified, largely comprising lipids and amino acids. Notable examples included PEP (16:0/18:1), octadecanedioic acid, 5,6-Epoxy-8,11,14-eicosatrienoic acid, 15,16-Dihydroxyoctadecanoic acid, and L-Tyrosine, all showing significant variation among breeds. Other endogenous compounds, such as palmitic amide, tetrahydrocortisone, and mesaconic acid, also displayed large response values (over 50-fold). Specifically, SQ exhibited the highest levels of octadecanedioic acid, PEP (16:0/18:1), 15,16-Dihydroxyoctadecanoic acid, and mesaconic acid; TR showed the highest abundance of 5,6-Epoxy-8,11,14-eicosatrienoic acid and palmitic amide; and WK had the highest levels of L-Tyrosine and tetrahydrocortisone. These compounds may serve as breed-specific biomarkers for authenticity identification.

### 3.5. Lipid Composition Analysis

In our previous study, lipids were identified as potential characteristic quality components of pigeon meat. Lipids also serve as one of the primary substrates for aroma generation in cooked meat products. It has been reported that up to 90% of aromatic substances in poultry meat originate from lipid reactions [32]. To further investigate the composition and characteristics of lipids in pigeon meat, lipidomics technology was employed.

**Figure 3:**
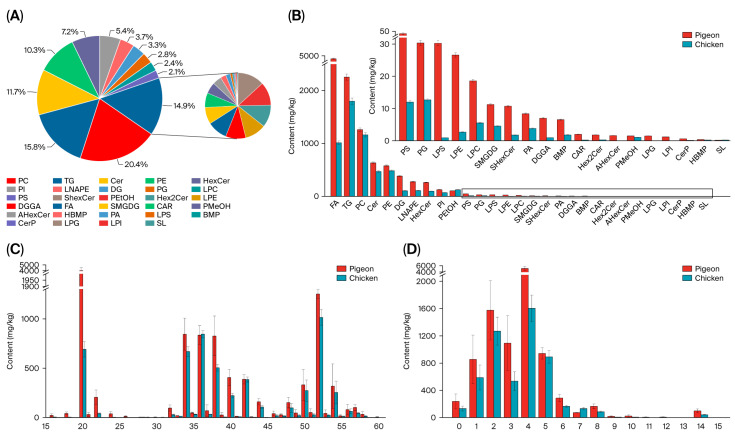
Composition and content of lipids in pigeon and chicken. (**A**) Composition of lipids in pigeon; (**B**) content of different types of lipids in pigeon and chicken; (**C**) content of lipids with different chain lengths in pigeon and chicken; (**D**) content of lipids with different degrees of unsaturation in pigeon and chicken.

A total of 1272 lipids were detected in pigeon and chicken meat, which were categorized into 29 subclasses (Figure 3A). Among these, phosphatidylcholine (PC), triglyceride (TG), ceramide (Cer), and phosphatidylethanolamine (PE) were the most abundant lipid classes. Previous studies have indicated that triglycerides and phospholipids are major contributors to lipid oxidation during meat processing, primarily due to the relationship between direct lipid oxidation and the degree of lipid unsaturation [33]. PC and PE, which are glycerophospholipids (GPs), contribute significantly to meat flavor. TG is the most abundant and important neutral lipid in meat and plays a critical role in cell growth and metabolism.

In terms of lipid content, pigeon meat contained significantly higher levels of total lipids compared to chicken meat. Specifically, lipids such as diglyceride (DG), fatty acid (FA), hexosylceramide (HexCer), phosphatidylinositol (PI), and phosphatidylserine (PS) showed significant differences in abundance (Figure 3B). Most of these belong to the categories of GPs and glycerolipids (GLs), suggesting that these are characteristic lipid components in pigeon meat.

Subsequently, the chain length and degree of unsaturation of the differential lipids were analyzed. The results revealed that lipids containing 20 carbon atoms were significantly more abundant in pigeon meat than in chicken (Figure 3C). Furthermore, lipids containing unsaturated bonds, especially those with four double bonds, were also higher in pigeon meat (Figure 3D). Both chain length and the unsaturation degree of lipids are closely associated with their biological functions, such as influencing cell membrane fluidity, modulating metabolic processes, and exerting pro-inflammatory effects [34].

To further investigate the distribution of and variation in lipids between pigeon and chicken, both unsupervised and supervised multivariate statistical analyses were applied. The data were preprocessed using log transformation and Pareto scaling. PCA revealed a clear separation trend between the two species within the first two principal components (Figure S1A), with PC1 and PC2 accounting for 49.8% and 13.6% of the total variance, respectively. The OPLS-DA score plot also showed distinct clustering between pigeon and chicken, consistent with the PCA results. The OPLS-DA model exhibited high explanatory power and predictive accuracy, with R^2^Y = 0.986 and Q^2^ = 0.84, indicating excellent model quality (Figure S1B).

**Figure 4:**
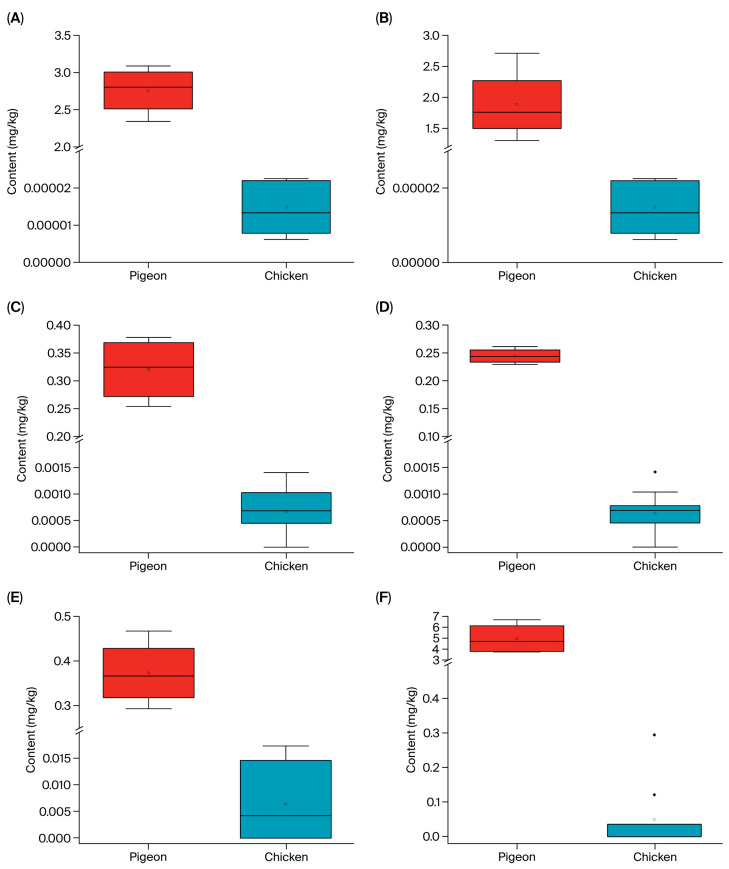
Content of lipid differential substances in pigeon and chicken. (**A**) Content of PS (18:0/20:4); (**B**) content of PE (16:2;O/2:0); (**C**) content of HexCer (9:0;2O/42:11); (**D**) content of Hex2Cer (38:1;2O); (**E**) content of PS (16:0;O/21:0); (**F**) content of PE (42:9).

Differential lipids were screened based on the criteria of |log_2_(fold change)| > 0.2, *p*-value < 0.05, and VIP > 1. A total of 213 differentially abundant lipids were identified, among which 135 were significantly more abundant in pigeon meat (Figure S1C). Notably, phosphatidylcholine (PC) and phosphatidylethanolamine (PE) emerged as the most prominent differential lipid classes. Six lipids—PS (18:0/20:4), PE (16:2; O/2:0), HexCer (9:0;2O/42:11), Hex2Cer (38:1;2O), PS (16:0; O/21:0), and PE (42:9)—were found at concentrations more than 50 times higher in pigeon than in chicken, suggesting their potential role as characteristic lipid components of pigeon meat (Figure 4A–F).

**Figure 5:**
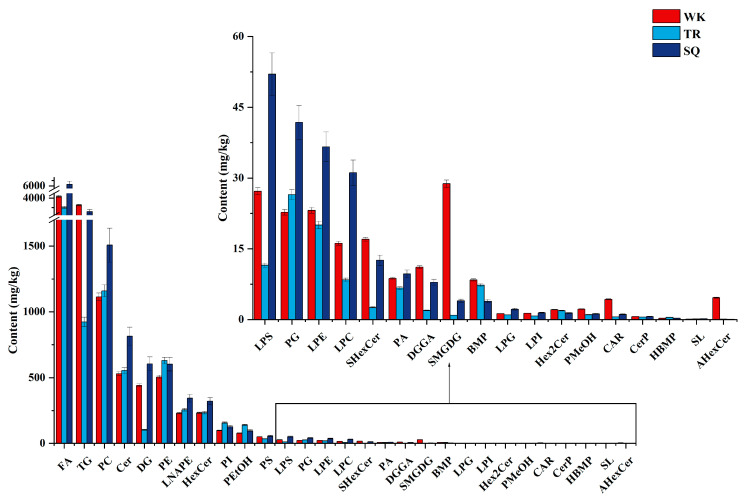
Content of different types of lipids in different breeds of pigeons.

Lipid profiles were further compared across different pigeon breeds. The total lipid content followed trends consistent with the fatty acid composition reported earlier in this study. Significant variations were also observed in lipid subclasses among the three breeds. For instance, SQ pigeon exhibited the highest levels of fatty acids (FA), phosphatidylcholine (PC), ceramide (Cer), hexosylceramide (HexCer), and N-acyl phosphatidylethanolamine (LNAPE). Tarim pigeon showed the highest abundance of phosphatidylethanolamine (PE) and phosphatidylethanol (PEtOH), whereas WK pigeon contained the highest levels of triglyceride (TG), monogalactosyldiacylglycerol (MGDG), and acylhexosylceramide (AHexCer) (Figure 5).

TG serves primarily as an energy reserve, while PC and PE play essential roles in intracellular transport, membrane fluidity, and metabolic regulation in muscle tissues [35]. Among the most abundant lipids, FA (20:4) was highest in the SQ pigeon, followed by WK and TR. Similar trends were observed for TG (16:0/18:0/18:2), TG (16:0/18:1/18:2), FA (20:3), FA (22:4), and lysophosphatidylserine (LPS (21:1)), among others (Table S2).

Notably, certain lipids exhibited extreme abundance variations—some exceeding thousand-fold differences—suggesting their potential use as breed-specific biomarkers for authenticity verification. For example, PE/Cer (15:3;2O/26:1; O) was over 3000 times more abundant in SQ pigeon than in the other two breeds, indicating it may serve as a characteristic marker for SQ identity.

Additionally, several lipids—including PC (18:3; O/20:4), PE (14:1; O/28:5), PEtOH (19:2/26:4), PC (11:0; O/28:5), DG (18:0; O/22:4), DG (17:1; O/22:4), Hex2Cer (18:0;2O/20:0), and Cer (22:3;2O/17:1)—showed no significant difference between SQ and TR pigeons, but were present at markedly higher responses compared to WK. These compounds may also aid in the detection of misrepresented WK products.

## 4. Conclusions

In the present study, the quality of pigeon meat was comprehensively evaluated through the analysis of conventional nutritional components integrated with multi-omics technologies. Poultry meat, particularly pigeon, was found to contain elevated levels of flavor-enhancing amino acids such as Arg and Ala, as well as ω-6 polyunsaturated fatty acids, including linoleic acid. Furthermore, pigeon meat exhibited a rich diversity and abundance of lipids, especially GPs and lipid species containing 20 carbon atoms or four unsaturated bonds. These components are likely to contribute to the umami taste of pigeon meat and may act as precursors for flavor formation during processing. Several endogenous compounds—including homovanillic acid, glycitin, tauroursodeoxycholic acid, and specific lipids such as PS (18:0_20:4), PE (16:2; O/2:0), HexCer (9:0;2O/42:11), Hex2Cer (38:1;2O), PS (16:0; O/21:0), and PE (42:9)—were identified as potential characteristic markers for authenticating pigeon meat. Additionally, significant compositional differences were observed among different pigeon breeds, reflected in the varying levels of compounds such as PEP (16:0/18:1), octadecanedioic acid, 5,6-Epoxy-8,11,14-eicosatrienoic acid, 15,16-Dihydroxyoctadecanoic acid, and L-Tyrosine. This study provides a comprehensive quality profile of pigeon meat and identifies key precursor compounds involved in the flavor development of processed pigeon products. These findings offer valuable insights for further research on pigeon meat and its derived products.

## Figures and Tables

**Table 1:** The content of amino acids and nucleotides in pigeon and chicken (mg/100 g).

		SQ (mg/100 g)	WK (mg/100 g)	TR (mg/100 g)	Chicken (mg/100 g)
Amino acids	Glu ^#^	27.70 ± 6.39 ^a^	27.06 ± 20.95 ^a^	12.46 ± 2.70 ^a^	14.27 ± 6.81 ^a^
	Ala ^#^	16.59 ± 3.42 ^a^	20.19 ± 7.04 ^a^	19.30 ± 3.88 ^a^	21.81 ± 3.71 ^a^
	Asp ^#^	7.98 ± 2.16 ^a^	9.95 ± 5.04 ^a^	9.59 ± 4.65 ^a^	12.08 ± 4.08 ^a^
	Gly ^#^	7.12 ± 1.51 ^a^	8.89 ± 2.54 ^a^	13.35 ± 5.86 ^a^	12.84 ± 1.73 ^a^
	Tyr ^#^	7.05 ± 1.47 ^a^	9.39 ± 7.73 ^a^	4.72 ± 2.03 ^a^	3.52 ± 1.20 ^a^
	Phe *^,#^	5.33 ± 1.12 ^a^	8.49 ± 4.09 ^a^	4.92 ± 1.69 ^a^	3.12 ± 1.30 ^a^
	Leu *	12.26 ± 2.33 ^a^	16.08 ± 6.81 ^a^	8.97 ± 3.56 ^a^	6.11 ± 2.47 ^a^
	Lys *	10.04 ± 3.18 ^a^	13.65 ± 6.78 ^a^	7.86 ± 1.92 ^a^	6.66 ± 3.12 ^a^
	Thr *	9.01 ± 2.25 ^a^	11.74 ± 3.59 ^a^	8.33 ± 1.61 ^a^	8.11 ± 2.52 ^a^
	Val *	7.99 ± 1.72 ^a^	10.78 ± 4.91 ^a^	5.63 ± 1.85 ^ab^	4.28 ± 1.28 ^b^
	Ile *	5.42 ± 1.18 ^a^	9.34 ± 3.9 ^a^	4.12 ± 1.53 ^ab^	2.64 ± 1.07 ^b^
	Met *	3.62 ± 0.69 ^a^	5.79 ± 2.07 ^a^	2.84 ± 1.75 ^ab^	1.64 ± 0.56 ^b^
	Ser	12.58 ± 2.48 ^a^	17.34 ± 5.42 ^a^	17.32 ± 6.19 ^a^	10.91 ± 5.56 ^a^
	Arg	10.55 ± 2.64 ^a^	13.47 ± 5.93 ^a^	8.63 ± 1.47 ^a^	8.19 ± 3.40 ^a^
	His	6.77 ± 1.67 ^a^	8.80 ± 4.09 ^a^	4.65 ± 1.04 ^a^	5.17 ± 1.18 ^a^
	Pro	5.40 ± 2.19 ^a^	5.92 ± 2.87 ^a^	4.66 ± 1.45 ^a^	5.32 ± 3.46 ^a^
	Essential amino acid	60.43 ± 12.76 ^a^	82.67 ± 35.34 ^a^	47.32 ± 13.74 ^ab^	37.76 ± 10.88 ^b^
	Umami amino acid	71.77 ± 14.58 ^a^	83.97 ± 42.69 ^a^	64.35 ± 14.27 ^a^	67.65 ± 12.37 ^a^
	Total	150.00 ± 30.72 ^a^	194.88 ± 87.79 ^a^	137.35 ± 26.88 ^a^	126.70 ± 30.63 ^a^
Nucleotides	IMP	124.76 ± 30.00 ^a^	49.26 ± 14.49 ^b^	86.83 ± 13.10 ^a^	49.92 ± 14.72 ^b^
	GMP	4.47 ± 0.26 ^a^	3.72 ± 1.84 ^a^	2.14 ± 2.35 ^a^	2.94 ± 2.29 ^a^
	AMP	6.12 ± 1.35 ^a^	3.97 ± 0.62 ^a^	2.57 ± 0.47 ^a^	2.76 ± 0.85 ^a^
	I	17.01 ± 4.52 ^c^	41.94 ± 11.11 ^a^	26.71 ± 4.05 ^b^	33.07 ± 6.64 ^b^
	Hx	7.76 ± 0.38 ^c^	10.77 ± 0.96 ^b^	15.23 ± 1.28 ^a^	10.12 ± 1.58 ^b^
	CMP	0.26 ± 0.07 ^c^	1.24 ± 0.20 ^a^	0.57 ± 0.08 ^b^	0.57 ± 0.02 ^b^
	UMP	0.72 ± 0.12 ^a^	0.35 ± 0.11 ^c^	0.56 ± 0.05 ^b^	0.55 ± 0.05 ^b^

Note: * represents essential amino acids; ^#^ represents umami amino acids; different letters indicate significant differences within different meats (*p* < 0.05).

## Data Availability

The original contributions presented in this study are included in the article/Supplementary Materials. Further inquiries can be directed to the corresponding authors.

## Supplementary Materials

The following supporting information can be downloaded at https://www.mdpi.com/article/10.3390/foods14183230/s1: Figure S1: Discrimination of pigeon and chicken; Table S1: Molecular weights and optimized MS parameters for flavored nucleotides in ESI+ mode; Table S2: Content of lipids in three breeds of pigeon (mg/kg).

## Abbreviations

The following abbreviations are used in this manuscript:

Glu	Glutamate
Ala	Alanine
Asp	Aspartate
Gly	Glycine
Tyr	Tyrosine
Phe	Phenylalanine
Leu	Leucine
Lys	Lysine
Thr	Threonine
Val	Valine
Ile	Isoleucine
Met	Methionine
Ser	Serine
Arg	Arginine
His	Histidine
Pro	Proline
IMP	Inosine monophosphate
GMP	Guanosine monophosphate
AMP	Adenosine monophosphate
I	Inosine
Hx	Hypoxanthine
CMP	Cytosine monophosphate
UMP	Uridine monophosphate
GP	Glycerophospholipid
FA	Fatty acid
TG	Triglyceride
PC	Phosphatidylcholine
Cer	Ceramide
PE	Phosphatidylethanolamine
DG	Diglyceride
LNAPE	N-Acylphosphatidylethanotamine
HexCer	Hexosylceramide
PI	Phosphatidylinositol
PEtOH	Phosphatidylethanol
PS	Phosphatidylserine
PG	Phosphatidylglycerol
LPS	Lyso-phosphatidylserine
LPE	Lyso-phosphatidylethanolamine
LPC	Lyso-phosphatidylcholine
SMGDG	Monogalactosyldiacylglycerol
PA	Phosphatidic acid
DGGA	Diacylglyceryl-α-D-glucuronide
BMP	Bis (monoacylglycero)phosphate
CAR	Constitutive Androstane Receptor
Hex2Cer	Hexosylceramides
PMeOH	Phosphatidylmethanol
LPG	Lyso-phosphatidylglycerol
LPI	Lyso-phosphatidylinositol
CerP	Ceramides phosphate
SL	Saccharolipid
C15:0	Pentadecanoic acid
C15:1	cis-10-Pentadecenoic acid
C16:0	Palmitic acid
C16:1	Palmitoleic acid
C17:0	Heptadecanoic acid
C18:0	Stearic acid
C18:1n9t	Elaidic acid
C18:1n9c	Oleic acid
C18:2n6t	Linolelaidic acid
C18:2n6c	Linoleic acid
C20:0	Arachidic acid
C18:3n6	γ-Linolenic acid
C20:1n9	cis-11-Eicosenoic acid
C18:3n3	α-Linolenic acid
C21:0	Heneicosanoic acid
C20:2	cis-11,14-Eicosadienoic acid
C22:0	Behenic acid
C20:3n6	cis-8,11,14-Eicosatrienoic acid
C22:1n9	Erucic acid
C20:3n3	cis-11,14,17-Eicasatrienoic acid
C20:4n6	Arachidonic acid
C23:0	Tricosanoic acid
C22:2n6	cis-13,16-Docosadienoic acid
C24:0	Lignoceric acid
C20:5n3	cis-5,8,11,14,17-Eicosapentaenoic acid
C24:1n9	Nervonic acid
C22:6n3	cis-4,7,10,13,16,19-Docosahexaenoic acid
