# Supplementary material for: Multi-Omics Characterization of Quality Attributes in Pigeon Meat
Source: Foods. 2025 Sep 17;14(18):3230. doi: 10.3390/foods14183230 (PMC12469855; doi:10.3390/foods14183230)
Supplement: Supplementary file 1 [file foods-14-03230-s001.zip › foods-3865049-supplementary.pdf]

Multi-Omics Characterization of Quality Attributes in Pigeon Meat

Xinran Wang [1], Yunyun Hu [2], Yan Liu [2], Cheng Li [1], Zheng Wang [3], Meiyu Liu [4], Jinhui Zhou [2,*] and Meng Wang [1,*]

[1] Institute of Quality Standard and Testing Technology, Beijing Academy of Agriculture and Forestry Sciences, Beijing 100097, China
[2] Institute of Food Science and Technology, Chinese Academy of Agricultural Sciences, Beijing 100193, China
[3] Institute of Animal Husbandry and Veterinary Medicine, Beijing Academy of Agriculture and Forestry Sciences, Beijing 100097, China
[4] School of Life Sciences and Food Engineering, Hebei University of Engineering, Handan 056000, China
* Correspondence: zhoujinhui@caas.cn (J.Z.); wangm@iqstt.cn (M.W.); Tel.: +86-010-62811838 (J.Z.); +86-010-81227735 (M.W.)

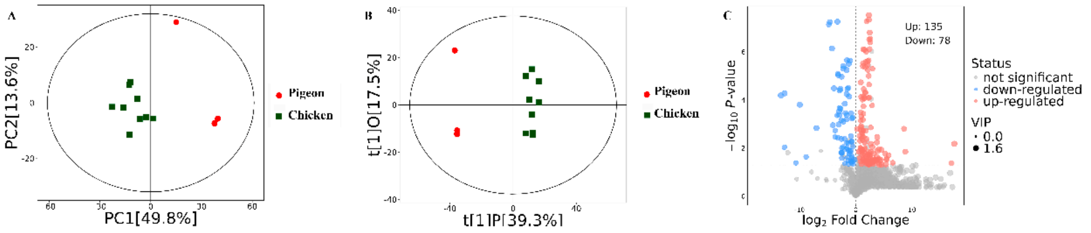

Figure S1 Discrimination of pigeon and chicken. A) PCA score plot of pigeon and chicken meat; (B) OPLS-DA score plot of pigeon and chicken meat; C) Volcano plot of all metabolites in pigeon and chicken meat.

Table S1: Molecular weights and optimized MS parameters for flavored nucleotides in ESI+ mode

Compound	Retention Time (min)	Precursor ions and Product ions (m/z)	CE (eV)
UMP	1.406	324.95/97.00*<br>324.95/213.05	15，9
GMP	2.037	364.20/152.20*<br>364.20/135.20	20，40
AMP	1.987	348.20/136.20*<br>348.20/119.15	20，53
I	3.630	269.20/137.15*<br>269.20/119.15	14，40
Hx	2.357	137.10/119.15*<br>137.10/110.15	24，24
IMP	1.965	349.30/137.20*<br>349.30/119.00	25，70
CMP	1.292	324.90/113.15*<br>324.90/112.00	21，15

Table S2: Content of lipids in three breeds of pigeon (mg/kg)

Compound name	Subclass	WK		TR		SQ	
		Average	SD	Average	SD	Average	SD
PC(18:3;O/20:4)	PC	0.0000	0.0000	0.6549	0.0260	0.8857	0.0762
PE(14:1;O/28:5)	PE	0.0000	0.0000	0.2822	0.0072	0.5742	0.0494
SMGDG(17:0;O/28:0)	SMGDG	0.0000	0.0000	0.0117	0.0003	0.3917	0.0337
PI(19:0;O/26:0)	PI	0.0000	0.0000	0.0118	0.0003	0.3911	0.0336
TG(18:1 ;O/16:1/18:1)	TG	0.0000	0.0000	0.0118	0.0003	0.3742	0.0322
PC(36:5;O/)	PC	0.0000	0.0000	0.0109	0.0003	0.3151	0.0271
TG(14:0;O/18:1/18:1)	TG	0.0000	0.0000	0.0103	0.0003	0.2072	0.0178
PC(11:0;O/28:5)	PC	0.0000	0.0000	0.0568	0.0015	0.1691	0.0146
DG(O/18:0;O/22:4)	DG	0.0000	0.0000	0.0359	0.0009	0.1554	0.0134
DG(17:1;O/22:4)	DG	0.0000	0.0000	0.0264	0.0007	0.1159	0.0100
PEtOH(19:2/26:4)	PEtOH	0.0000	0.0000	0.1637	0.0042	0.0476	0.0041
PE/Cer(15:3;2O/26:1;O)	Cer	0.0000	0.0000	0.0000	0.0000	0.0432	0.0037
Hex2Cer(18:0;2O/20:0)	Hex2Cer	0.0000	0.0000	0.0435	0.0011	0.0318	0.0027
Cer(22:3;2O/17:1)	Cer	0.0000	0.0000	0.0236	0.0006	0.0249	0.0021
TG(18:0;O/14:1/14:1)	TG	0.0000	0.0000	0.0009	0.0000	0.0160	0.0014
PC(39:4)	PC	0.0000	0.0000	0.0047	0.0001	0.0132	0.0011
LNAPE(16:1/N/26:3)	LNAPE	0.0000	0.0000	0.0000	0.0000	0.0074	0.0006
PMeOH(22:4/22:6)	PMeOH	0.0000	0.0000	0.0460	0.0012	0.0071	0.0006
DGGA(26:0/24:2)	DGGA	0.0000	0.0000	0.0022	0.0001	0.0007	0.0001

PI(18:1/18:1;2O)	PI	0.0017	0.0000	0.0000	0.0000	0.0811	0.0070
PI(16:0/18:1;3O)	PI	0.0026	0.0001	0.0035	0.0001	0.0890	0.0077
TG(22:4;O/18:0/18:0)	TG	0.0086	0.0002	0.0045	0.0001	0.2941	0.0253
Cer(21:0;2O/22:4;(3OH)(FA 20:4))	Cer	0.0001	0.0000	0.0001	0.0000	0.0035	0.0003
TG(20:4;O/18:0/18:0)	TG	0.0432	0.0011	0.0222	0.0006	1.1565	0.0995
Hex2Cer(16:3;2O/15:1)	Hex2Cer	0.0069	0.0002	0.0000	0.0000	0.1708	0.0147
PC(12:0;O/28:4)	PC	0.1009	0.0025	0.6109	0.0157	2.1794	0.1875
DGGA(15:1/22:5)	DGGA	0.0132	0.0003	0.0000	0.0000	0.2835	0.0244
PE/Cer(20:1;2O/26:7)	Cer	0.0692	0.0017	0.1620	0.0042	1.2415	0.1068
PC(16:2;O/2:0)	PC	0.0262	0.0007	0.0974	0.0025	0.4232	0.0364
PE(21:2;O/20:3)	PE	0.0021	0.0001	0.0000	0.0000	0.0312	0.0027
PC(41:6)	PC	0.0010	0.0000	0.0000	0.0000	0.0143	0.0012
PI/Cer(12:0;2O/24:1;O)	Cer	0.0110	0.0003	0.0000	0.0000	0.1558	0.0134
PC(19:0/18:2)	PC	0.3045	0.0076	1.9835	0.0509	4.1061	0.3533
TG(16:0;O/18:0/22:4)	TG	0.0373	0.0009	0.0119	0.0003	0.4951	0.0426
PC(48:11)	PC	0.0003	0.0000	0.0000	0.0000	0.0043	0.0004
LPC(40:4)	LPC	0.0367	0.0009	0.1576	0.0040	0.4823	0.0415
SHexCer(37:2;3O)	SHexCer	0.0169	0.0004	0.0035	0.0001	0.2210	0.0190
PE(18:0/20:4;3O)	PE	0.2493	0.0062	0.0011	0.0000	3.1192	0.2684
DGGA(18:1/20:5)	DGGA	0.1043	0.0026	0.0000	0.0000	1.2945	0.1114
PE(18:0/20:3;3O)	PE	0.0684	0.0017	0.0000	0.0000	0.8466	0.0728
PC(18:0;O/22:4)	PC	0.0487	0.0012	0.1291	0.0033	0.5975	0.0514

LPC(38:8)	LPC	0.0436	0.0011	0.0000	0.0000	0.5284	0.0455
DG(18:1/26:7)	DG	0.0550	0.0014	0.1835	0.0047	0.6471	0.0557
TG(14:1;O/16:0/16:0)	TG	0.0077	0.0002	0.0077	0.0002	0.0904	0.0078
TG(20:3;O/18:1/18:1)	TG	0.2138	0.0053	0.0243	0.0006	2.4149	0.2078
SHexCer(15:1;2O/24:2)	SHexCer	0.0017	0.0000	0.0006	0.0000	0.0189	0.0016
PC(18:0;O/20:4)	PC	2.6219	0.0655	3.7371	0.0960	29.3531	2.5257
TG(16:1;O/16:0/16:0)	TG	0.1925	0.0048	0.1979	0.0051	2.0927	0.1801
HexCer(8:1;2O/36:10)	HexCer	0.3752	0.0094	0.0036	0.0001	4.0103	0.3451
PI(18:0/22:3)	PI	0.1192	0.0030	1.3792	0.0354	1.2536	0.1079
PC(12:0;O/28:5)	PC	1.1389	0.0285	1.4691	0.0377	11.9017	1.0241
TG(16:0;O/18:0/18:1)	TG	0.8041	0.0201	0.7060	0.0181	8.3716	0.7203
DGGA(22:3/16:4)	DGGA	0.1242	0.0031	0.0000	0.0000	1.2244	0.1054
SHexCer(14:0;2O/21:1)	SHexCer	0.0325	0.0008	0.0000	0.0000	0.3200	0.0275
PC(10:0;O/28:3)	PC	0.1504	0.0038	0.4620	0.0119	1.4005	0.1205
TG(18:0;O/18:1/18:2)	TG	0.5471	0.0137	0.1900	0.0049	4.9357	0.4247
TG(18:1;O/16:0/18:0)	TG	0.2746	0.0069	0.2022	0.0052	2.4695	0.2125
PC(27:3)	PC	0.0177	0.0004	0.0000	0.0000	0.1589	0.0137
PC(18:0;O/18:1)	PC	0.0390	0.0010	0.1929	0.0050	0.3496	0.0301
PE(24:4;O/17:0)	PE	0.0081	0.0002	0.0380	0.0010	0.0718	0.0062
PE/Cer(13:0;2O/24:0;O)	Cer	1.3047	0.0326	4.8906	0.1256	11.6073	0.9988
Cer(26:0;2O/16:1;(2OH))	Cer	0.0019	0.0000	0.0149	0.0004	0.0167	0.0014
PC(11:0;O/28:7)	PC	0.0388	0.0010	0.0176	0.0005	0.3318	0.0286

LPC(34:4)	LPC	0.0065	0.0002	0.0036	0.0001	0.0544	0.0047
TG(18:1;O/16:0/16:0)	TG	1.3337	0.0333	0.9653	0.0248	11.1797	0.9620
DG(16:0;O/22:4)	DG	0.0688	0.0017	0.0899	0.0023	0.5356	0.0461
SMGDG(12:0;O/28:7)	SMGDG	0.0194	0.0005	0.0000	0.0000	0.1466	0.0126
TG(18:0;O/18:1/18:1)	TG	0.1343	0.0034	0.0714	0.0018	1.0014	0.0862
TG(18:0;O/18:0/18:1)	TG	0.1802	0.0045	0.0977	0.0025	1.3024	0.1121
PC(37:1)	PC	0.0367	0.0009	0.0072	0.0002	0.2649	0.0228
DGGA(22:2/16:4)	DGGA	0.1544	0.0039	0.0035	0.0001	1.0860	0.0934
PE(18:0/20:5;3O)	PE	0.4546	0.0114	0.0006	0.0000	3.1301	0.2693
PI(15:0/18:1(d7))	PI	0.0111	0.0003	0.0007	0.0000	0.0761	0.0065
PI/Cer(13:2;2O/24:2;O)	Cer	1.4407	0.0360	0.0000	0.0000	9.8188	0.8449
TG(16:2;O/18:0/18:0)	TG	1.5839	0.0396	0.7596	0.0195	10.5838	0.9107
DGGA(27:0/13:1)	DGGA	0.0055	0.0001	0.0000	0.0000	0.0366	0.0032
PC(16:0/18:1;O)	PC	0.1119	0.0028	0.0005	0.0000	0.7425	0.0639
PE(18:0/20:4;2O)	PE	0.1197	0.0030	0.0027	0.0001	0.7926	0.0682
PC(16:0;O/22:3)	PC	0.1638	0.0041	0.6376	0.0164	1.0761	0.0926
PI/Cer(13:2;2O/24:0;O)	Cer	5.8993	0.1475	0.1985	0.0051	38.3788	3.3024
TG(18:1/18:1/22:0)	TG	0.0015	0.0000	0.0004	0.0000	0.0097	0.0008
TG(16:0;O/16:0/22:5)	TG	0.4090	0.0102	0.1064	0.0027	2.5663	0.2208
PC(27:4)	PC	0.0118	0.0003	0.0003	0.0000	0.0735	0.0063
TG(20:2/20:4/20:4)	TG	0.0414	0.0010	0.0627	0.0016	0.2570	0.0221
PE/Cer(12:2;2O/18:5)	Cer	1.0422	0.0261	0.8508	0.0218	6.4399	0.5541

TG(13:0/15:0/15:0)	TG	0.0038	0.0001	0.0040	0.0001	0.0233	0.0020
FA(22:3)	FA	4.5518	0.1138	6.7947	0.1745	27.3978	2.3575
PC(19:0/18:1)	PC	0.1101	0.0028	0.3326	0.0085	0.6563	0.0565
PE(38:3)	PE	8.5305	0.2133	12.0838	0.3103	50.3362	4.3313
PC(10:0;O/28:4)	PC	5.6499	0.1412	10.3040	0.2646	33.3030	2.8656
DG(18:1/22:3)	DG	0.7023	0.0176	0.7996	0.0205	4.1339	0.3557
PC(16:0/18:2;O)	PC	0.2783	0.0070	0.0076	0.0002	1.6270	0.1400
LPC(20:4;O/)	LPC	0.0395	0.0010	0.0925	0.0024	0.2249	0.0194
PI(24:4/18:5)	PI	0.0034	0.0001	0.0469	0.0012	0.0189	0.0016
PC(36:5)	PC	0.0124	0.0003	0.0000	0.0000	0.0694	0.0060
LPC(18:4;O/)	LPC	0.0235	0.0006	0.0101	0.0003	0.1289	0.0111
PC(25:1)	PC	0.0057	0.0001	0.0012	0.0000	0.0311	0.0027
PI(18:0/18:1;O)	PI	0.1477	0.0037	0.0012	0.0000	0.8031	0.0691
PE/Cer(12:0;2O/20:1)	Cer	0.0602	0.0015	0.0779	0.0020	0.3252	0.0280
PI/Cer(17:3;2O/26:0;O)	Cer	0.0123	0.0003	0.0000	0.0000	0.0659	0.0057
PE(16:0/34:1)	PE	0.0473	0.0012	0.0036	0.0001	0.2520	0.0217
PI/Cer(14:0;2O/25:0;O)	Cer	0.0495	0.0012	0.0498	0.0013	0.2639	0.0227
DG(18:1/36:2)	DG	0.2032	0.0051	0.0612	0.0016	1.0761	0.0926
PC(16:4/32:9)	PC	0.0012	0.0000	0.0000	0.0000	0.0061	0.0005
PI(16:4/18:4)	PI	0.0084	0.0002	0.0648	0.0017	0.0435	0.0037
PC(18:0/18:1;3O)	PC	0.2806	0.0070	0.0000	0.0000	1.4411	0.1240
LNAPE(18:0/N/16:0)	LNAPE	0.3058	0.0076	0.0269	0.0007	1.5684	0.1350

TG(18:1;O/16:1/16:1)	TG	0.1650	0.0041	0.0963	0.0025	0.8437	0.0726
PC(16:0/20:4;3O)	PC	0.1903	0.0048	0.0031	0.0001	0.9607	0.0827
TG(8:0/16:0/16:1)	TG	0.0482	0.0012	0.0219	0.0006	0.2409	0.0207
PC(16:1;O/20:3)	PC	1.8248	0.0456	2.5270	0.0649	9.1114	0.7840
PS(10:0/26:1)	PS	0.7790	0.0195	0.0576	0.0015	3.8894	0.3347
PI(18:0/16:1)	PI	0.3711	0.0093	1.7008	0.0437	1.8448	0.1587
PI(13:1;O/26:5)	PI	0.0462	0.0012	0.0000	0.0000	0.2268	0.0195
HexCer(10:0;2O/44:9)	HexCer	0.1657	0.0041	0.0508	0.0013	0.8117	0.0698
PC(18:0/18:2;O)	PC	1.7151	0.0429	0.0339	0.0009	8.3503	0.7185
PC(18:1/18:2;O)	PC	0.1292	0.0032	0.0047	0.0001	0.6278	0.0540
PG(16:0/20:0)	PG	1.0632	0.0266	1.9766	0.0507	5.1304	0.4414
LPC(40:5)	LPC	1.1369	0.0284	1.1153	0.0286	5.4812	0.4716
PE(35:4)	PE	0.1409	0.0035	0.0090	0.0002	0.6790	0.0584
PEtOH(18:1/26:4)	PEtOH	0.1135	0.0028	0.5089	0.0131	0.5422	0.0467
LNAPE(18:2/N/20:0)	LNAPE	2.6020	0.0650	3.5088	0.0901	12.4225	1.0689
HexCer(12:1;3O/24:2;(2OH))	HexCer	0.7649	0.0191	3.2774	0.0841	3.5940	0.3093
PE(18:2;O/20:1)	PE	0.3656	0.0091	2.0218	0.0519	1.7101	0.1471
TG(16:0;O/16:0/16:0)	TG	0.2778	0.0069	0.3286	0.0084	1.2738	0.1096
Cer(15:1;2O/24:1)	Cer	0.0003	0.0000	0.0000	0.0000	0.0015	0.0001
PC(18:0;O/22:5)	PC	1.1512	0.0288	0.9561	0.0245	5.2154	0.4488
HexCer(17:3;2O/44:12)	HexCer	0.0975	0.0024	0.0271	0.0007	0.4404	0.0379
PC(18:1/18:2;2O)	PC	0.3722	0.0093	0.0028	0.0001	1.6798	0.1445

TG(18:1/18:1/18:1)	TG	0.2012	0.0050	0.5120	0.0131	0.9057	0.0779
SMGDG(13:1;O/28:2)	SMGDG	0.0047	0.0001	0.0000	0.0000	0.0211	0.0018
LNAPE(18:0/N/18:0)	LNAPE	0.0919	0.0023	0.0000	0.0000	0.4099	0.0353
PI/Cer(15:3;2O/22:2;O)	Cer	1.6723	0.0418	0.0000	0.0000	7.4492	0.6410
TG(14:0;O/16:0/16:0)	TG	0.0133	0.0003	0.0135	0.0003	0.0583	0.0050
PE/Cer(18:2;2O/26:7)	Cer	2.5920	0.0648	1.1056	0.0284	11.2052	0.9642
PE(18:2;O/22:4)	PE	6.8212	0.1705	11.7798	0.3025	29.4746	2.5362
PI(16:2;O/18:5)	PI	0.0030	0.0001	0.0024	0.0001	0.0131	0.0011
SHexCer(15:1;2O/22:2)	SHexCer	0.1966	0.0049	0.0058	0.0001	0.8357	0.0719
DG(18:1/20:1)	DG	0.1682	0.0042	0.0664	0.0017	0.7041	0.0606
LPC(40:9)	LPC	0.1510	0.0038	0.0066	0.0002	0.6276	0.0540
PI/Cer(15:2;2O/26:0;O)	Cer	0.0696	0.0017	0.0000	0.0000	0.2884	0.0248
FA(20:3)	FA	93.8161	2.3454	59.6473	1.5315	388.7813	33.4533
PC(15:0/18:1(d7))	PC	0.5287	0.0132	1.7065	0.0438	2.1905	0.1885
PE/Cer(12:2;2O/26:1;O)	Cer	0.3171	0.0079	1.0190	0.0262	1.3105	0.1128
PG(17:1;O/26:6)	PG	0.3178	0.0079	0.0156	0.0004	1.3125	0.1129
PG(26:7;O/19:0)	PG	0.0996	0.0025	0.1596	0.0041	0.4098	0.0353
TG(18:2/18:2/20:2)	TG	3.7285	0.0932	2.5992	0.0667	15.2952	1.3161
PC(32:2;O/)	PC	0.1660	0.0041	0.7869	0.0202	0.6803	0.0585
TG(22:4;O/16:0/16:0)	TG	0.4639	0.0116	0.0901	0.0023	1.8957	0.1631
PC(18:1/18:1;3O)	PC	0.2572	0.0064	0.0003	0.0000	1.0507	0.0904
Cer(14:0;2O/40:2;O(FA 16:0))	Cer	0.0007	0.0000	0.0003	0.0000	0.0029	0.0002

PE(19:2/19:2)	PE	0.2952	0.0074	1.1607	0.0298	1.1992	0.1032
PC(11:0;O/28:3)	PC	0.1606	0.0040	0.7056	0.0181	0.6497	0.0559
SMGDG(22:6;O/28:6)	SMGDG	0.0093	0.0002	0.0110	0.0003	0.0376	0.0032
PE(20:2;O/18:1)	PE	0.8192	0.0205	2.0115	0.0516	3.2850	0.2827
PE(41:9)	PE	0.3515	0.0088	0.0036	0.0001	1.4091	0.1212
PG(16:0/21:0)	PG	0.0289	0.0007	0.0866	0.0022	0.1155	0.0099
PI/Cer(13:2;2O/26:0;O)	Cer	9.3974	0.2349	0.4274	0.0110	37.4379	3.2214
PS(16:0/20:4)	PS	1.3355	0.0334	0.3413	0.0088	5.3149	0.4573
PC(36:0)	PC	0.2237	0.0056	0.4185	0.0107	0.8902	0.0766
PC(16:2/16:2)	PC	0.0368	0.0009	0.0027	0.0001	0.1459	0.0126
DG(18:1/24:1)	DG	0.0447	0.0011	0.0785	0.0020	0.1763	0.0152
LPE(20:3)	LPE	0.2799	0.0070	0.3274	0.0084	1.0990	0.0946
PG(15:1;O/26:5)	PG	0.0083	0.0002	0.0452	0.0012	0.0324	0.0028
DG(22:1/18:2)	DG	0.1075	0.0027	0.1188	0.0031	0.4162	0.0358
PC(23:1)	PC	0.0112	0.0003	0.0000	0.0000	0.0433	0.0037
PC(36:2;O/)	PC	0.1421	0.0036	0.0188	0.0005	0.5463	0.0470
SMGDG(9:0;O/22:0)	SMGDG	0.1183	0.0030	0.0073	0.0002	0.4517	0.0389
PC(30:1;O/)	PC	0.0038	0.0001	0.0118	0.0003	0.0145	0.0013
PC(16:2;O/4:0)	PC	0.0044	0.0001	0.0015	0.0000	0.0167	0.0014
LPC(40:8)	LPC	0.0567	0.0014	0.0010	0.0000	0.2127	0.0183
PC(18:0/20:2)	PC	0.4189	0.0105	1.0637	0.0273	1.5698	0.1351
HexCer(19:3;2O/44:12)	HexCer	0.0919	0.0023	0.0000	0.0000	0.3405	0.0293

PE(18:0/20:5;O)	PE	0.4333	0.0108	0.0337	0.0009	1.5982	0.1375
PI(18:0/20:4;3O)	PI	0.1369	0.0034	0.0003	0.0000	0.5033	0.0433
DG(20:0/18:1)	DG	0.4878	0.0122	0.4941	0.0127	1.7904	0.1541
TG(18:0;O/16:0/18:0)	TG	0.1615	0.0040	0.1085	0.0028	0.5914	0.0509
PC(16:3;O/20:0)	PC	6.1586	0.1540	6.8702	0.1764	22.4676	1.9333
PE(18:0/20:4;O)	PE	1.0873	0.0272	0.0815	0.0021	3.9345	0.3386
PC(32:1;O/)	PC	0.2273	0.0057	0.9579	0.0246	0.8140	0.0700
PC(16:4/6:0)	PC	0.2369	0.0059	0.0518	0.0013	0.8479	0.0730
PI(16:4/20:5)	PI	0.1739	0.0043	0.1591	0.0041	0.6179	0.0532
PC(37:0;O/)	PC	0.0316	0.0008	0.0224	0.0006	0.1111	0.0096
PC(35:4)	PC	0.1246	0.0031	0.1122	0.0029	0.4380	0.0377
PC(18:1;O/20:4)	PC	8.2047	0.2051	7.1429	0.1834	28.6605	2.4661
PI/Cer(24:0;2O/25:0;O)	Cer	11.0075	0.2752	0.7620	0.0196	38.2887	3.2946
PE(16:3;O/4:0)	PE	0.3622	0.0091	0.4353	0.0112	1.2551	0.1080
PE(36:3)	PE	0.3182	0.0080	0.0354	0.0009	1.0977	0.0945
TG(20:1/20:4/20:4)	TG	0.0542	0.0014	0.0589	0.0015	0.1870	0.0161
PI(16:0/20:3)	PI	0.1163	0.0029	0.5097	0.0131	0.3974	0.0342
PC(18:1/20:4;3O)	PC	0.0738	0.0018	0.0000	0.0000	0.2512	0.0216
HexCer(14:0;2O/44:9)	HexCer	0.4893	0.0122	0.0218	0.0006	1.6648	0.1433
PI(18:0/20:3)	PI	10.6580	0.2664	56.8485	1.4596	36.2359	3.1180
PC(18:0;O/18:2)	PC	3.0544	0.0764	6.0370	0.1550	10.3799	0.8932
PC(16:1;O/18:1)	PC	8.1002	0.2025	30.0399	0.7713	27.3564	2.3539

PI(18:0/22:5)	PI	1.7916	0.0448	2.1710	0.0557	6.0030	0.5165
PC(16:0/20:3;3O)	PC	0.1930	0.0048	0.0000	0.0000	0.6452	0.0555
Hex2Cer(31:3;2O)	Hex2Cer	0.0403	0.0010	0.1416	0.0036	0.1340	0.0115
DG(18:0/18:1)	DG	12.7893	0.3197	10.4783	0.2690	41.9898	3.6131
Cer(14:0;2O/18:4;(3OH)(FA 20:4))	Cer	0.0301	0.0008	0.0427	0.0011	0.0985	0.0085
DG(18:0/18:0)	DG	0.1132	0.0028	0.2269	0.0058	0.3677	0.0316
SHexCer(14:0;2O/36:0)	SHexCer	2.0189	0.0505	0.1539	0.0040	6.4745	0.5571
PS(10:0/26:4)	PS	0.2013	0.0050	0.2208	0.0057	0.6417	0.0552
PC(18:1;O/16:0)	PC	1.4930	0.0373	2.0647	0.0530	4.7466	0.4084
PE(18:3;O/22:6)	PE	0.1429	0.0036	0.3681	0.0095	0.4532	0.0390
PC(10:0;O/28:5)	PC	2.6332	0.0658	2.2534	0.0579	8.3505	0.7185
PI(14:1/26:2)	PI	0.0093	0.0002	0.0280	0.0007	0.0295	0.0025
PI(18:0/18:2)	PI	0.0842	0.0021	0.0000	0.0000	0.2651	0.0228
PE(20:4/20:5)	PE	0.1216	0.0030	0.0680	0.0017	0.3821	0.0329
DG(18:1/22:1)	DG	0.1173	0.0029	0.0489	0.0013	0.3675	0.0316
HexCer(14:3;2O/32:9)	HexCer	3.4192	0.0855	0.1114	0.0029	10.7023	0.9209
PE/Cer(20:1;2O/26:4)	Cer	2.9313	0.0733	8.4801	0.2177	9.0474	0.7785
PC(18:0/20:4;2O)	PC	0.2875	0.0072	0.0000	0.0000	0.8852	0.0762
PC(16:0/20:4;4O)	PC	0.2450	0.0061	0.0000	0.0000	0.7485	0.0644
PS(24:2/16:4)	PS	0.1037	0.0026	0.3782	0.0097	0.3162	0.0272
PI/Cer(14:0;2O/25:1;O)	Cer	0.0970	0.0024	0.0000	0.0000	0.2934	0.0252
PC(16:0/20:4;O)	PC	0.1499	0.0037	0.0066	0.0002	0.4511	0.0388

PC(21:1)	PC	0.0027	0.0001	0.0000	0.0000	0.0079	0.0007
HexCer(12:0;2O/44:9)	HexCer	0.6618	0.0165	0.0607	0.0016	1.9584	0.1685
LPC(34:0)	LPC	0.9246	0.0231	1.4458	0.0371	2.7327	0.2351
PC(16:4;O/22:5)	PC	0.0126	0.0003	0.1141	0.0029	0.0372	0.0032
LPC(36:4)	LPC	0.1574	0.0039	0.3204	0.0082	0.4622	0.0398
PI/Cer(25:0;2O/26:0;O)	Cer	4.7963	0.1199	0.2324	0.0060	14.0425	1.2083
PE/Cer(14:3;2O/26:1;O)	Cer	10.4814	0.2620	30.2476	0.7766	30.6655	2.6387
PI(18:0/20:4;O)	PI	0.0427	0.0011	0.0004	0.0000	0.1248	0.0107
SHexCer(14:0;2O/38:0)	SHexCer	0.7989	0.0200	0.0422	0.0011	2.3303	0.2005
PI/Cer(12:0;2O/25:0;O)	Cer	7.1594	0.1790	3.8213	0.0981	20.7301	1.7838
PC(18:1;O/22:4)	PC	1.1295	0.0282	1.5669	0.0402	3.2402	0.2788
DGGA(16:0/22:6)	DGGA	0.5318	0.0133	0.0009	0.0000	1.5246	0.1312
PC(36:1;O/)	PC	0.2914	0.0073	0.3777	0.0097	0.8345	0.0718
PE(18:1;O/22:5)	PE	3.3533	0.0838	7.6961	0.1976	9.5277	0.8198
PC(16:0;O/22:6)	PC	13.9827	0.3496	7.7869	0.1999	39.1673	3.3702
PI(18:1/18:2)	PI	0.7377	0.0184	0.9559	0.0245	2.0548	0.1768
Cer(8:0;2O/38:8)	Cer	0.0280	0.0007	0.0696	0.0018	0.0778	0.0067
DG(16:0/16:1)	DG	15.8935	0.3973	10.4339	0.2679	44.0988	3.7945
PI(16:0;O/19:0)	PI	3.9573	0.0989	2.1834	0.0561	10.9671	0.9437
PEtOH(16:0/22:5)	PEtOH	0.1480	0.0037	0.3215	0.0083	0.4101	0.0353
LPE(16:1)	LPE	0.0693	0.0017	0.1221	0.0031	0.1910	0.0164
SMGDG(16:3;O/26:6)	SMGDG	0.1678	0.0042	0.0005	0.0000	0.4603	0.0396

HexCer(13:1;3O/22:2;(2OH))	HexCer	0.1562	0.0039	0.0000	0.0000	0.4275	0.0368
PC(16:0/18:3;O)	PC	1.3520	0.0338	0.0579	0.0015	3.6985	0.3182
DG(18:0/22:5)	DG	1.1720	0.0293	0.3017	0.0077	3.2022	0.2755
PE/Cer(23:3;2O/26:5)	Cer	0.1919	0.0048	0.0000	0.0000	0.5210	0.0448
PE(18:2;O/18:2)	PE	8.4124	0.2103	23.6223	0.6065	22.7883	1.9609
PC(47:8)	PC	0.0266	0.0007	0.0278	0.0007	0.0717	0.0062
HBMP(12:0/14:1/16:2)	HBMP	0.0127	0.0003	0.0210	0.0005	0.0339	0.0029
PC(48:0)	PC	0.0077	0.0002	0.0057	0.0001	0.0206	0.0018
PI/Cer(12:0;2O/25:1;O)	Cer	1.1897	0.0297	0.1859	0.0048	3.1704	0.2728
PI/Cer(13:2;2O/26:2;O)	Cer	1.5745	0.0394	0.0028	0.0001	4.1853	0.3601
PE(16:0/20:4;O)	PE	0.1422	0.0036	0.0086	0.0002	0.3743	0.0322
PC(36:3)	PC	0.1242	0.0031	0.0029	0.0001	0.3267	0.0281
PC(37:2)	PC	1.3529	0.0338	2.3189	0.0595	3.5574	0.3061
PI/Cer(13:2;2O/24:1;O)	Cer	7.7085	0.1927	0.1737	0.0045	20.2155	1.7395
PC(18:1;O/22:5)	PC	0.9521	0.0238	0.6397	0.0164	2.4627	0.2119
PC(16:0/18:1;3O)	PC	0.2989	0.0075	0.0000	0.0000	0.7728	0.0665
PC(13:1;O/3:0)	PC	0.1085	0.0027	0.1243	0.0032	0.2802	0.0241
PEtOH(22:3/26:4)	PEtOH	0.3524	0.0088	0.9111	0.0234	0.9058	0.0779
DGGA(14:0/15:1)	DGGA	0.0153	0.0004	0.0013	0.0000	0.0393	0.0034
Cer(18:1;2O/23:0)	Cer	0.3095	0.0077	0.5125	0.0132	0.7875	0.0678
PMeOH(26:2/20:3)	PMeOH	0.0665	0.0017	0.2237	0.0057	0.1688	0.0145
PE(22:0/18:1)	PE	0.5502	0.0138	1.0793	0.0277	1.3955	0.1201

PE(34:1/22:4)	PE	0.6049	0.0151	0.0566	0.0015	1.5253	0.1312
HexCer(8:0;2O/34:7)	HexCer	0.1812	0.0045	0.3034	0.0078	0.4566	0.0393
TG(18:1/18:2/22:6)	TG	0.2210	0.0055	0.1678	0.0043	0.5550	0.0478
PC(15:0/20:4)	PC	1.2452	0.0311	0.4613	0.0118	3.1255	0.2689
PC(34:3)	PC	0.1338	0.0033	0.0028	0.0001	0.3354	0.0289
PC(16:0/20:5;3O)	PC	0.0620	0.0015	0.0000	0.0000	0.1553	0.0134
PE(18:0/20:5)	PE	0.9144	0.0229	0.0828	0.0021	2.2906	0.1971
PG(20:0/14:1)	PG	7.9510	0.1988	9.6448	0.2476	19.8550	1.7085
PI(10:0;O/26:7)	PI	0.0492	0.0012	0.1435	0.0037	0.1218	0.0105
Hex2Cer(29:1;2O)	Hex2Cer	0.0430	0.0011	0.0000	0.0000	0.1064	0.0092
LNAPE(16:0/N/20:0)	LNAPE	0.1428	0.0036	0.1803	0.0046	0.3527	0.0303
DG(22:0/18:1)	DG	0.2233	0.0056	0.3960	0.0102	0.5500	0.0473
PI(14:0;O/17:0)	PI	0.0447	0.0011	0.0034	0.0001	0.1098	0.0094
LNAPE(20:3/N/18:0)	LNAPE	11.6417	0.2910	13.8203	0.3548	28.4339	2.4466
LPC(20:4)	LPC	2.7793	0.0695	0.8027	0.0206	6.7544	0.5812
PC(14:1/20:2)	PC	0.1241	0.0031	0.1134	0.0029	0.3006	0.0259
LPE(19:0)	LPE	0.1365	0.0034	0.1172	0.0030	0.3301	0.0284
DG(17:0/18:1)	DG	0.6453	0.0161	0.2970	0.0076	1.5534	0.1337
PC(16:2;O/22:6)	PC	0.0235	0.0006	0.0005	0.0000	0.0562	0.0048
FA(16:3)	FA	4.0482	0.1012	0.7090	0.0182	9.6663	0.8318
PE(18:1/32:1)	PE	0.1917	0.0048	0.0272	0.0007	0.4571	0.0393
DG(18:2;O/22:4)	DG	0.0666	0.0017	0.0452	0.0012	0.1585	0.0136

PE/Cer(16:0;2O/26:4)	Cer	22.4147	0.5604	28.5477	0.7330	52.4217	4.5107
PI(22:0;O/26:0)	PI	0.0568	0.0014	0.0005	0.0000	0.1328	0.0114
PC(40:3)	PC	0.4576	0.0114	1.1099	0.0285	1.0685	0.0919
HexCer(20:0;2O/38:10)	HexCer	0.7555	0.0189	0.0390	0.0010	1.7631	0.1517
PI(18:1/18:1;O)	PI	0.0662	0.0017	0.0019	0.0000	0.1545	0.0133
PI(18:1/20:4;3O)	PI	0.0762	0.0019	0.0000	0.0000	0.1776	0.0153
SMGDG(O/22:3;O/28:6)	SMGDG	0.0093	0.0002	0.0059	0.0002	0.0216	0.0019
DGGA(18:5/22:6)	DGGA	0.0880	0.0022	0.1837	0.0047	0.2044	0.0176
PC(35:4;O)	PC	1.5726	0.0393	4.4547	0.1144	3.6499	0.3141
PG(16:0/18:0;O)	PG	0.0288	0.0007	0.0649	0.0017	0.0668	0.0057
PC(22:0/18:2)	PC	0.5447	0.0136	1.3172	0.0338	1.2625	0.1086
PC(16:0;O/16:0)	PC	7.0077	0.1752	8.9584	0.2300	16.1972	1.3937
PE(18:1;O/22:3)	PE	1.7775	0.0444	5.5476	0.1424	4.0987	0.3527
PE(16:1;O/22:0)	PE	0.1357	0.0034	0.1114	0.0029	0.3109	0.0267
PC(16:4;O/4:0)	PC	2.4252	0.0606	0.7576	0.0195	5.5541	0.4779
PC(41:7)	PC	0.0096	0.0002	0.0000	0.0000	0.0219	0.0019
PC(16:2;O/22:5)	PC	1.9675	0.0492	1.0347	0.0266	4.4392	0.3820
LNAPE(18:0/N/20:5)	LNAPE	0.0111	0.0003	0.0363	0.0009	0.0251	0.0022
HexCer(12:0;2O/44:10)	HexCer	1.0391	0.0260	0.2089	0.0054	2.3249	0.2001
PE(20:0;O/18:2)	PE	0.5264	0.0132	0.7859	0.0202	1.1766	0.1012
PC(32:0;O)	PC	2.1545	0.0539	2.5406	0.0652	4.8104	0.4139
PE(16:0;O/16:0)	PE	0.0880	0.0022	0.3817	0.0098	0.1953	0.0168

HexCer(38:2;3O/15:1;(2OH))	HexCer	0.2553	0.0064	0.1106	0.0028	0.5648	0.0486
DG(18:1/22:5)	DG	1.6328	0.0408	0.4058	0.0104	3.6059	0.3103
PC(10:0;O/28:7)	PC	3.5560	0.0889	1.9130	0.0491	7.8300	0.6737
PC(13:1;O/5:0)	PC	2.2987	0.0575	2.3825	0.0612	5.0465	0.4342
PI(18:0/16:2)	PI	0.6639	0.0166	0.7550	0.0194	1.4562	0.1253
PC(24:0)	PC	0.0090	0.0002	0.0001	0.0000	0.0196	0.0017
PC(18:0;O/16:0)	PC	1.5905	0.0398	3.9532	0.1015	3.4794	0.2994
PC(16:1;O/16:0)	PC	5.7266	0.1432	7.8577	0.2018	12.5192	1.0772
LPC(36:2)	LPC	0.0693	0.0017	0.2255	0.0058	0.1513	0.0130
Cer(18:1;2O/21:0)	Cer	0.1776	0.0044	0.2732	0.0070	0.3874	0.0333
SMGDG(9:0;O/26:1)	SMGDG	0.5647	0.0141	0.2478	0.0064	1.2257	0.1055
PC(18:0/20:3)	PC	11.5219	0.2880	18.2565	0.4687	24.9071	2.1432
PC(35:0;O)	PC	0.1229	0.0031	0.1743	0.0045	0.2647	0.0228
Cer(18:1;2O/20:0)	Cer	11.3128	0.2828	5.5149	0.1416	24.3674	2.0967
PC(35:5)	PC	0.0786	0.0020	0.0291	0.0007	0.1692	0.0146
LNAPE(18:0/N/20:4)	LNAPE	0.0249	0.0006	0.0217	0.0006	0.0535	0.0046
DG(16:0/22:5)	DG	3.1038	0.0776	1.3351	0.0343	6.6650	0.5735
PE(10:0;O/10:0)	PE	0.1012	0.0025	0.0257	0.0007	0.2169	0.0187
PC(33:4)	PC	0.0611	0.0015	0.0009	0.0000	0.1309	0.0113
PE(12:0;O/26:4)	PE	5.0897	0.1272	11.0000	0.2824	10.8602	0.9345
PE/Cer(18:2;2O/26:3)	Cer	45.0542	1.1264	104.4428	2.6816	95.6108	8.2270
PS(25:0/13:1)	PS	0.2725	0.0068	0.4851	0.0125	0.5742	0.0494

Cer(18:1;2O/22:0)	Cer	1.1919	0.0298	1.2330	0.0317	2.5108	0.2160
PE(18:1;O/16:0)	PE	2.1834	0.0546	3.2458	0.0833	4.5893	0.3949
PC(16:0;O/18:1)	PC	13.1015	0.3275	30.5178	0.7836	27.5086	2.3670
Cer(18:1;2O/24:0)	Cer	1.7428	0.0436	3.0160	0.0774	3.6582	0.3148
PE(16:0/36:1)	PE	0.0519	0.0013	0.0005	0.0000	0.1082	0.0093
PI(10:0;O/24:4)	PI	0.2917	0.0073	0.4288	0.0110	0.6074	0.0523
PE(18:1/36:1)	PE	0.0498	0.0012	0.0043	0.0001	0.1033	0.0089
HexCer(9:0;2O/24:4)	HexCer	0.0339	0.0008	0.0321	0.0008	0.0701	0.0060
DG(19:0;O/18:1)	DG	0.4863	0.0122	0.9391	0.0241	1.0047	0.0864
LPC(16:1)	LPC	0.4535	0.0113	0.5838	0.0150	0.9336	0.0803
SL(19:0;O/17:2)	SL	0.0998	0.0025	0.1718	0.0044	0.2050	0.0176
LPC(17:1;O)	LPC	0.0542	0.0014	0.0121	0.0003	0.1106	0.0095
TG(18:0/18:1/20:0)	TG	0.6259	0.0156	0.1787	0.0046	1.2706	0.1093
PE(42:8)	PE	0.1497	0.0037	0.3466	0.0089	0.3029	0.0261
PC(18:5/22:6)	PC	0.0072	0.0002	0.0071	0.0002	0.0145	0.0013
PC(14:0;O/17:0)	PC	0.3083	0.0077	0.1987	0.0051	0.6234	0.0536
LPE(20:0)	LPE	0.2207	0.0055	0.0990	0.0025	0.4457	0.0383
SHexCer(14:0;2O/40:0)	SHexCer	0.0532	0.0013	0.0099	0.0003	0.1070	0.0092
PC(18:2;O/22:6)	PC	0.1644	0.0041	0.0968	0.0025	0.3302	0.0284
HexCer(9:0;2O/24:3)	HexCer	0.8009	0.0200	0.6207	0.0159	1.6040	0.1380
PC(16:0;O/20:4)	PC	1.9676	0.0492	1.0573	0.0271	3.9046	0.3360
PC(16:4;O/3:0)	PC	0.0935	0.0023	0.0546	0.0014	0.1853	0.0159

HexCer(9:0;2O/22:6)	HexCer	0.1729	0.0043	0.1212	0.0031	0.3423	0.0295
PE(18:0;O/20:4)	PE	8.2439	0.2061	22.7173	0.5833	16.3215	1.4044
PE(20:1;O/16:0)	PE	0.3431	0.0086	0.4575	0.0117	0.6788	0.0584
PC(38:5)	PC	0.0324	0.0008	0.0000	0.0000	0.0638	0.0055
Cer(18:1;2O/25:0)	Cer	0.0269	0.0007	0.1275	0.0033	0.0528	0.0045
LPE(40:6)	LPE	1.7797	0.0445	3.0042	0.0771	3.4818	0.2996
HexCer(16:3;2O/19:1;O)	HexCer	0.1386	0.0035	0.1135	0.0029	0.2711	0.0233
DGGA(26:2/16:4)	DGGA	0.1226	0.0031	0.0990	0.0025	0.2390	0.0206
LPS(21:1)	LPS	26.4113	0.6603	11.4186	0.2932	51.4672	4.4286
PG(21:1/22:5)	PG	0.1464	0.0037	0.0821	0.0021	0.2852	0.0245
TG(16:0/17:0/18:1)	TG	0.1816	0.0045	0.2507	0.0064	0.3522	0.0303
TG(18:1/18:1/22:4)	TG	1.6259	0.0406	0.7618	0.0196	3.1368	0.2699
Hex2Cer(14:0;2O)	Hex2Cer	0.0028	0.0001	0.0038	0.0001	0.0054	0.0005
LPC(18:1;O)	LPC	0.3873	0.0097	0.2889	0.0074	0.7433	0.0640
FA(16:2)	FA	13.6066	0.3402	6.4615	0.1659	26.1061	2.2463
PI(14:1/22:6)	PI	0.0389	0.0010	0.0000	0.0000	0.0744	0.0064
PE(12:0;O/28:4)	PE	1.2765	0.0319	2.2808	0.0586	2.4151	0.2078
LPE(20:4)	LPE	8.4059	0.2101	5.6037	0.1439	15.7803	1.3578
LNAPE(17:0/N/22:4)	LNAPE	0.0134	0.0003	0.0054	0.0001	0.0251	0.0022
Cer(8:0;2O/34:4)	Cer	0.9992	0.0250	0.5853	0.0150	1.8584	0.1599
PE(19:0;O/20:4)	PE	0.2667	0.0067	0.3908	0.0100	0.4949	0.0426
PG(16:1/18:2)	PG	0.0875	0.0022	0.2583	0.0066	0.1617	0.0139

TG(18:1/18:1/19:1)	TG	1.0431	0.0261	1.7055	0.0438	1.9268	0.1658
PC(16:1;O/0:4)	PC	14.7714	0.3693	8.6658	0.2225	27.1104	2.3328
TG(17:1/18:1/18:1)	TG	0.6612	0.0165	1.0708	0.0275	1.2129	0.1044
PC(26:3)	PC	0.0156	0.0004	0.0000	0.0000	0.0286	0.0025
DG(18:1/18:2)	DG	39.1566	0.9789	8.0329	0.2062	71.6571	6.1658
PI(18:1/20:3;2O)	PI	0.1429	0.0036	0.0005	0.0000	0.2592	0.0223
PC(18:0/20:4;O)	PC	0.2556	0.0064	0.0103	0.0003	0.4616	0.0397
SHexCer(14:0;2O/20:1)	SHexCer	0.0744	0.0019	0.0191	0.0005	0.1344	0.0116
PC(44:0/20:1)	PC	0.0144	0.0004	0.0343	0.0009	0.0258	0.0022
PE/Cer(17:3;2O/25:1;O)	Cer	3.3343	0.0834	3.1510	0.0809	5.9660	0.5134
TG(16:0;O/18:2/18:2)	TG	0.5085	0.0127	0.0771	0.0020	0.9095	0.0783
PS(16:0;O/21:0)	PS	3.7615	0.0940	3.8307	0.0984	6.7198	0.5782
PEtOH(16:1/22:3)	PEtOH	0.2990	0.0075	0.4906	0.0126	0.5328	0.0458
PC(16:3;O/4:0)	PC	0.1731	0.0043	0.1096	0.0028	0.3084	0.0265
PE/Cer(14:3;2O/26:2;O)	Cer	4.7339	0.1183	6.4846	0.1665	8.3886	0.7218
PE(16:1/18:2)	PE	0.1794	0.0045	0.7271	0.0187	0.3140	0.0270
LNAPE(18:2/N/19:0)	LNAPE	0.2433	0.0061	0.3115	0.0080	0.4253	0.0366
PG(18:2/18:2)	PG	0.0981	0.0025	0.0241	0.0006	0.1714	0.0147
HBMP(12:0/14:0/16:0)	HBMP	0.0576	0.0014	0.0553	0.0014	0.1004	0.0086
HexCer(12:0;2O/44:11)	HexCer	0.1731	0.0043	0.0508	0.0013	0.2999	0.0258
PG(18:1/18:3;O)	PG	0.0500	0.0012	0.1132	0.0029	0.0866	0.0075
PS(18:0/20:3)	PS	1.9778	0.0494	2.0305	0.0521	3.4265	0.2948

FA(26:4)	FA	0.5072	0.0127	0.3336	0.0086	0.8777	0.0755
PI/Cer(12:1;2O/21:1;O)	Cer	0.4114	0.0103	0.1546	0.0040	0.7090	0.0610
PE(22:6;O/18:2)	PE	0.2554	0.0064	0.2514	0.0065	0.4388	0.0378
LPG(18:2)	LPG	1.2828	0.0321	0.9994	0.0257	2.1996	0.1893
PC(37:2;O)	PC	0.1572	0.0039	0.2795	0.0072	0.2691	0.0232
TG(18:1/18:1/22:2)	TG	0.5483	0.0137	0.1852	0.0048	0.9354	0.0805
PC(16:1;O/18:3)	PC	0.2345	0.0059	0.1277	0.0033	0.3994	0.0344
Cer(10:0;3O/23:0;(2OH))	Cer	0.0061	0.0002	0.0000	0.0000	0.0104	0.0009
HexCer(16:0;2O/44:11)	HexCer	0.4650	0.0116	0.0288	0.0007	0.7915	0.0681
PC(35:5;O)	PC	0.0103	0.0003	0.0000	0.0000	0.0174	0.0015
PC(18:1;O/18:2)	PC	1.4360	0.0359	0.9693	0.0249	2.4326	0.2093
TG(18:1/18:2/22:4)	TG	1.4482	0.0362	0.6864	0.0176	2.4513	0.2109
FA(24:3)	FA	1.6068	0.0402	0.6705	0.0172	2.7193	0.2340
PC(34:0;O)	PC	0.0825	0.0021	0.1908	0.0049	0.1393	0.0120
PC(42:5)	PC	0.1679	0.0042	0.1796	0.0046	0.2829	0.0243
PC(33:2;O)	PC	0.1674	0.0042	0.4548	0.0117	0.2808	0.0242
PI/Cer(23:2;2O/26:0;O)	Cer	5.0195	0.1255	0.2693	0.0069	8.3991	0.7227
TG(8:0/16:0/16:0)	TG	0.0885	0.0022	0.0352	0.0009	0.1471	0.0127
LNAPE(20:3/N/24:2)	LNAPE	0.1032	0.0026	0.1321	0.0034	0.1714	0.0147
DGGA(14:0/17:2)	DGGA	0.1437	0.0036	0.3149	0.0081	0.2385	0.0205
PC(16:0;O/18:2)	PC	23.9172	0.5979	31.1680	0.8003	39.5262	3.4011
Hex2Cer(14:1;2O)	Hex2Cer	0.0026	0.0001	0.0012	0.0000	0.0042	0.0004

PS(14:0/26:4)	PS	1.1590	0.0290	1.0863	0.0279	1.9076	0.1641
PC(18:5;O/18:0)	PC	0.9736	0.0243	1.8810	0.0483	1.5982	0.1375
PC(18:1;O/22:6)	PC	0.9548	0.0239	0.3560	0.0091	1.5667	0.1348
HexCer(19:3;3O/38:2;(2OH))	HexCer	0.2718	0.0068	0.0144	0.0004	0.4433	0.0381
PE(18:0/20:3;4O)	PE	0.0634	0.0016	0.0711	0.0018	0.1032	0.0089
TG(18:1/18:1/21:0)	TG	1.1441	0.0286	0.6824	0.0175	1.8620	0.1602
PE(18:1;O/22:4)	PE	22.9925	0.5748	33.2763	0.8544	37.3617	3.2148
CAR(17:3)	CAR	0.0993	0.0025	0.0815	0.0021	0.1607	0.0138
HexCer(9:1;2O/28:7)	HexCer	0.0173	0.0004	0.0000	0.0000	0.0279	0.0024
PC(36:3;O)	PC	3.1110	0.0778	3.2586	0.0837	5.0137	0.4314
SHexCer(14:0;2O/36:1)	SHexCer	0.2256	0.0056	0.0115	0.0003	0.3634	0.0313
PC(34:2;O)	PC	20.1522	0.5038	20.8605	0.5356	32.2900	2.7784
PC(20:5;O/17:0)	PC	0.0928	0.0023	0.1999	0.0051	0.1486	0.0128
LPC(18:3)	LPC	0.0968	0.0024	0.0017	0.0000	0.1548	0.0133
PE(42:9)	PE	0.2933	0.0073	0.3431	0.0088	0.4674	0.0402
Cer(14:0;2O/31:0;(2OH))	Cer	0.0004	0.0000	0.0002	0.0000	0.0006	0.0001
Cer(18:1;2O/26:7)	Cer	0.1607	0.0040	0.0007	0.0000	0.2558	0.0220
PC(16:0;O/18:0)	PC	0.1347	0.0034	0.2182	0.0056	0.2143	0.0184
HexCer(15:3;2O/22:6)	HexCer	1.3462	0.0337	2.2894	0.0588	2.1415	0.1843
LPE(18:2)	LPE	3.6426	0.0911	2.7877	0.0716	5.7825	0.4976
TG(18:0/18:1/20:1)	TG	0.7084	0.0177	0.9408	0.0242	1.1200	0.0964
LPE(16:1;O)	LPE	0.4109	0.0103	0.3218	0.0083	0.6485	0.0558

PI(18:1/20:4;O)	PI	0.0196	0.0005	0.0033	0.0001	0.0309	0.0027
PC(16:1;O/18:2)	PC	37.2019	0.9300	37.7343	0.9689	58.4693	5.0311
FA(26:5)	FA	6.4559	0.1614	2.0997	0.0539	10.1182	0.8706
HexCer(8:0;2O/32:8)	HexCer	118.9792	2.9745	124.6742	3.2011	185.9739	16.0024
PE(26:3/17:0)	PE	0.0154	0.0004	0.0319	0.0008	0.0241	0.0021
PC(40:1)	PC	0.1356	0.0034	0.3843	0.0099	0.2110	0.0182
PC(34:3;O)	PC	47.2042	1.1801	48.8715	1.2548	73.4489	6.3200
PI/Cer(25:1;2O/26:0;O)	Cer	3.4594	0.0865	0.1786	0.0046	5.3814	0.4630
PE(18:1/18:2;2O)	PE	0.0502	0.0013	0.0726	0.0019	0.0780	0.0067
PC(18:2;O/18:2)	PC	10.7220	0.2681	8.9403	0.2295	16.6620	1.4337
LNAPE(20:4/N/18:0)	LNAPE	166.9149	4.1729	172.6290	4.4324	259.0444	22.2899
PC(19:2/19:2)	PC	0.0983	0.0025	0.0004	0.0000	0.1525	0.0131
LPC(18:2;O)	LPC	0.2875	0.0072	0.1070	0.0027	0.4415	0.0380
PG(24:5;O/17:0)	PG	0.6823	0.0171	0.9217	0.0237	1.0476	0.0901
PE(10:0;O/8:0)	PE	20.2823	0.5071	14.8440	0.3811	31.0476	2.6715
LPE(20:1)	LPE	0.2452	0.0061	0.1546	0.0040	0.3748	0.0323
PE(18:1/20:5;O)	PE	0.1221	0.0031	0.0433	0.0011	0.1856	0.0160
TG(18:0/18:1/22:4)	TG	1.8504	0.0463	0.4466	0.0115	2.8087	0.2417
PE(16:0;O/18:0)	PE	0.3814	0.0095	0.4765	0.0122	0.5754	0.0495
HexCer(12:0;2O/44:8)	HexCer	0.0424	0.0011	0.0923	0.0024	0.0637	0.0055
PC(32:1)	PC	7.3723	0.1843	15.9125	0.4086	11.0645	0.9521
PE(15:0/18:1(d7))	PE	0.8282	0.0207	1.2872	0.0330	1.2420	0.1069

PE(18:0;O/16:0)	PE	0.8121	0.0203	0.8988	0.0231	1.2119	0.1043
PG(21:2;O/26:7)	PG	0.1741	0.0044	0.2871	0.0074	0.2585	0.0222
PE(16:2;O/2:0)	PE	1.8265	0.0457	1.3008	0.0334	2.7098	0.2332
DG(18:2/20:2)	DG	1.5763	0.0394	0.4772	0.0123	2.3372	0.2011
FA(20:4)	FA	3562.1047	89.0526	2511.3850	64.4815	5279.9394	454.3204
LPE(22:0)	LPE	0.0249	0.0006	0.0299	0.0008	0.0369	0.0032
PE(13:1;O/5:0)	PE	0.0550	0.0014	0.1436	0.0037	0.0812	0.0070
PI/Cer(21:1;2O/26:0;O)	Cer	7.5027	0.1876	0.2629	0.0068	11.0674	0.9523
PEtOH(18:2/24:4)	PEtOH	1.5554	0.0389	1.8276	0.0469	2.2891	0.1970
HexCer(8:0;2O/30:6)	HexCer	46.3375	1.1584	60.5327	1.5542	68.1080	5.8605
PC(18:1/20:4;O)	PC	0.1317	0.0033	0.0020	0.0001	0.1934	0.0166
FA(24:5)	FA	13.4943	0.3374	3.6770	0.0944	19.7351	1.6981
PC(18:0/22:4)	PC	8.8718	0.2218	12.5237	0.3216	12.8901	1.1091
LPC(16:1;O)	LPC	0.2823	0.0071	0.1627	0.0042	0.4067	0.0350
PE/Cer(15:3;2O/24:2;O)	Cer	0.3612	0.0090	0.0142	0.0004	0.5191	0.0447
LPE(20:2;O)	LPE	0.0741	0.0019	0.0439	0.0011	0.1064	0.0092
PG(18:5;O/22:6)	PG	0.0146	0.0004	0.0003	0.0000	0.0210	0.0018
PC(16:0/20:1)	PC	2.6749	0.0669	2.5849	0.0664	3.8372	0.3302
PS(14:1/24:2)	PS	2.4309	0.0608	2.0126	0.0517	3.4854	0.2999
PC(37:5;O)	PC	1.0094	0.0252	1.2891	0.0331	1.4390	0.1238
LPC(20:1)	LPC	0.2557	0.0064	0.0884	0.0023	0.3635	0.0313
PC(19:0;O/7:0)	PC	0.0815	0.0020	0.0880	0.0023	0.1155	0.0099

LPC(18:0;O)	LPC	0.2256	0.0056	0.0938	0.0024	0.3195	0.0275
TG(18:2/18:3/20:3)	TG	1.6682	0.0417	0.5671	0.0146	2.3592	0.2030
PC(34:0)	PC	13.1513	0.3288	8.8345	0.2268	18.5168	1.5933
FA(22:4)	FA	179.1953	4.4799	133.3308	3.4234	251.8224	21.6684
PE/Cer(13:2;2O/16:4)	Cer	2.7845	0.0696	1.0263	0.0264	3.8858	0.3344
PC(34:1)	PC	0.0824	0.0021	0.0954	0.0025	0.1149	0.0099
PE/Cer(21:3;2O/26:2;O)	Cer	12.0629	0.3016	11.8865	0.3052	16.7902	1.4447
PE(8:0;O/28:5)	PE	0.5851	0.0146	0.9441	0.0242	0.8141	0.0700
HexCer(16:0;2O/17:0;O)	HexCer	0.0688	0.0017	0.0852	0.0022	0.0956	0.0082
PE(16:1;O/16:1)	PE	0.1393	0.0035	0.8754	0.0225	0.1931	0.0166
PE(16:4;O/6:0)	PE	0.1597	0.0040	0.0947	0.0024	0.2207	0.0190
Cer(25:0;2O/15:1)	Cer	1.8964	0.0474	0.8428	0.0216	2.6203	0.2255
Hex2Cer(27:0;2O)	Hex2Cer	0.0962	0.0024	0.3834	0.0098	0.1328	0.0114
PC(18:0/22:5)	PC	2.0797	0.0520	3.0381	0.0780	2.8678	0.2468
PI(13:1;O/22:6)	PI	0.2510	0.0063	0.4341	0.0111	0.3445	0.0296
HexCer(9:0;2O/30:4)	HexCer	0.0317	0.0008	0.0428	0.0011	0.0433	0.0037
PI(18:0;O/18:2)	PI	3.7017	0.0925	4.4275	0.1137	5.0486	0.4344
Hex2Cer(32:6;2O)	Hex2Cer	0.0121	0.0003	0.0093	0.0002	0.0165	0.0014
Cer(21:0;2O/44:9)	Cer	0.0170	0.0004	0.0255	0.0007	0.0232	0.0020
PE(16:1;O/18:3)	PE	0.2401	0.0060	0.3074	0.0079	0.3258	0.0280
HexCer(13:1;3O/20:2;(2OH))	HexCer	1.8294	0.0457	0.0013	0.0000	2.4658	0.2122
PE(22:4;O/16:1)	PE	0.0724	0.0018	0.0959	0.0025	0.0976	0.0084

TG(16:0/18:1/26:5)	TG	0.3694	0.0092	0.0835	0.0021	0.4954	0.0426
PE/Cer(13:1;2O/22:6)	Cer	1.0623	0.0266	1.6025	0.0411	1.4148	0.1217
PI(22:4;O/16:0)	PI	0.4354	0.0109	0.2140	0.0055	0.5794	0.0499
LPC(40:10)	LPC	0.1174	0.0029	0.0642	0.0016	0.1562	0.0134
TG(17:0/19:1/19:1)	TG	0.4409	0.0110	0.3886	0.0100	0.5861	0.0504
HexCer(13:1;3O/24:2;(2OH))	HexCer	0.0102	0.0003	0.0249	0.0006	0.0136	0.0012
PE(32:1/22:4)	PE	1.3475	0.0337	0.1142	0.0029	1.7802	0.1532
LPC(36:6)	LPC	5.1582	0.1290	1.7771	0.0456	6.7875	0.5840
PI(22:3;O/26:5)	PI	1.6000	0.0400	0.7889	0.0203	2.1031	0.1810
HexCer(8:1;2O/30:6)	HexCer	0.1061	0.0027	0.2508	0.0064	0.1394	0.0120
TG(18:1/18:1/19:0)	TG	3.4319	0.0858	2.2989	0.0590	4.4994	0.3872
PE(16:0/18:0)	PE	0.4109	0.0103	0.6235	0.0160	0.5379	0.0463
PC(40:9)	PC	0.0384	0.0010	0.0031	0.0001	0.0501	0.0043
HexCer(14:0;2O/44:11)	HexCer	2.4815	0.0620	0.2003	0.0051	3.2342	0.2783
DG(17:1/18:1)	DG	2.5557	0.0639	0.4388	0.0113	3.3172	0.2854
PI(13:1;O/26:1)	PI	0.0173	0.0004	0.0017	0.0000	0.0224	0.0019
Cer(18:1;2O/26:1)	Cer	0.0238	0.0006	0.0613	0.0016	0.0308	0.0026
LPE(22:4)	LPE	0.5954	0.0149	0.2438	0.0063	0.7675	0.0660
LNAPE(16:0/N/20:5)	LNAPE	0.1165	0.0029	0.2863	0.0074	0.1498	0.0129
HexCer(16:0;2O/23:0;O)	HexCer	0.3314	0.0083	0.3121	0.0080	0.4258	0.0366
DGGA(16:2/20:4)	DGGA	0.5205	0.0130	0.4230	0.0109	0.6684	0.0575
Cer(8:1;2O/5:0)	Cer	0.7175	0.0179	0.6007	0.0154	0.9199	0.0792

HexCer(8:0;2O/32:6)	HexCer	5.9775	0.1494	6.5080	0.1671	7.6561	0.6588
LNAPE(20:4/N/16:0)	LNAPE	0.3073	0.0077	0.0118	0.0003	0.3930	0.0338
TG(17:1/18:1/18:2)	TG	0.5412	0.0135	0.5326	0.0137	0.6914	0.0595
PEtOH(18:1/24:4)	PEtOH	69.2872	1.7322	131.0033	3.3636	88.5275	7.6175
TG(16:0/18:0/18:2)	TG	355.8854	8.8971	144.4294	3.7083	454.1790	39.0805
PC(18:2;O/20:3)	PC	5.8390	0.1460	7.4109	0.1903	7.4335	0.6396
PE(22:4;O/17:0)	PE	0.1534	0.0038	0.1682	0.0043	0.1953	0.0168
PC(34:4;O)	PC	0.3283	0.0082	0.1229	0.0032	0.4175	0.0359
PE(18:5;O/2:0)	PE	0.6517	0.0163	0.2672	0.0069	0.8268	0.0711
LNAPE(22:4/N/24:2)	LNAPE	0.2090	0.0052	0.1738	0.0045	0.2647	0.0228
TG(15:0/15:0/15:0)	TG	0.0618	0.0015	0.0271	0.0007	0.0779	0.0067
DG(16:1/18:1)	DG	290.8776	7.2719	54.0963	1.3890	366.2201	31.5120
PC(18:0/18:2)	PC	227.0228	5.6756	241.7644	6.2075	285.4293	24.5602
Cer(18:0;2O/16:0)	Cer	0.2397	0.0060	0.1358	0.0035	0.3009	0.0259
FA(26:6)	FA	6.9565	0.1739	0.7712	0.0198	8.6868	0.7475
LNAPE(18:2/N/18:0)	LNAPE	0.6299	0.0157	1.0349	0.0266	0.7840	0.0675
TG(16:0/16:0/18:1)	TG	212.0134	5.3003	83.7916	2.1514	263.6693	22.6878
PC(43:8)	PC	0.0127	0.0003	0.0167	0.0004	0.0157	0.0014
PC(16:1/18:2)	PC	6.6728	0.1668	13.5381	0.3476	8.2834	0.7128
PC(16:4;O/20:2)	PC	0.2041	0.0051	0.2095	0.0054	0.2529	0.0218
FA(28:7)	FA	2.8658	0.0716	2.9134	0.0748	3.5516	0.3056
LPE(18:1;O)	LPE	4.5917	0.1148	6.3186	0.1622	5.6809	0.4888

PA(18:0/26:4)	PA	5.7757	0.1444	3.9098	0.1004	7.1441	0.6147
PE/Cer(12:2;2O/26:2;O)	Cer	4.8319	0.1208	8.5101	0.2185	5.9586	0.5127
PG(17:2;O/26:7)	PG	0.3051	0.0076	0.4059	0.0104	0.3761	0.0324
PC(35:1)	PC	1.6387	0.0410	2.6868	0.0690	2.0178	0.1736
TG(16:0/16:0/16:1)	TG	11.2071	0.2802	5.7977	0.1489	13.7981	1.1873
TG(13:1/21:0/21:0)	TG	0.5834	0.0146	0.4004	0.0103	0.7168	0.0617
PG(16:0/18:2)	PG	1.4333	0.0358	0.8486	0.0218	1.7579	0.1513
PE(16:0;O/18:2)	PE	1.6134	0.0403	3.8504	0.0989	1.9742	0.1699
Cer(18:0;2O/20:0)	Cer	0.0770	0.0019	0.0660	0.0017	0.0941	0.0081
PC(16:1;O/20:5)	PC	1.9196	0.0480	0.5701	0.0146	2.3325	0.2007
Cer(23:2;2O/22:5)	Cer	0.0299	0.0007	0.0000	0.0000	0.0363	0.0031
SMGDG(8:0;O/28:2)	SMGDG	0.2947	0.0074	0.3328	0.0085	0.3575	0.0308
PE(18:2;O/20:4)	PE	11.4250	0.2856	20.4461	0.5250	13.8349	1.1904
LNAPE(16:0/N/18:0)	LNAPE	0.5154	0.0129	0.6710	0.0172	0.6237	0.0537
Cer(26:0;2O/17:2)	Cer	0.0205	0.0005	0.0317	0.0008	0.0248	0.0021
PI(10:0;O/24:3)	PI	0.0893	0.0022	0.0695	0.0018	0.1079	0.0093
PC(36:4;O)	PC	6.6471	0.1662	5.3358	0.1370	8.0261	0.6906
PE(32:1/22:5)	PE	1.0252	0.0256	0.1015	0.0026	1.2373	0.1065
TG(18:1;O/20:1/20:1)	TG	0.0440	0.0011	0.0007	0.0000	0.0530	0.0046
PC(18:4;O/18:2)	PC	1.1927	0.0298	1.5402	0.0395	1.4368	0.1236
PC(35:3)	PC	0.0015	0.0000	0.0016	0.0000	0.0018	0.0002
SMGDG(9:0;O/24:0)	SMGDG	0.5148	0.0129	0.1574	0.0040	0.6180	0.0532

PG(21:0;O/22:1)	PG	1.5891	0.0397	1.7509	0.0450	1.9042	0.1638
HexCer(8:0;2O/30:5)	HexCer	0.1816	0.0045	0.2192	0.0056	0.2171	0.0187
Cer(18:1;2O/24:1)	Cer	3.4011	0.0850	2.5201	0.0647	4.0576	0.3491
SMGDG(8:0;O/26:1)	SMGDG	0.1089	0.0027	0.0172	0.0004	0.1299	0.0112
HexCer(21:0;2O/19:1;O)	HexCer	2.2653	0.0566	2.5039	0.0643	2.6980	0.2322
PE(12:0;O/28:5)	PE	1.2555	0.0314	1.3186	0.0339	1.4889	0.1281
TG(18:1/18:1/20:3)	TG	14.5828	0.3646	4.3499	0.1117	17.2534	1.4846
Cer(18:0;2O/18:0)	Cer	0.1011	0.0025	0.1982	0.0051	0.1194	0.0103
PG(23:0;O/18:0)	PG	1.2130	0.0303	0.7347	0.0189	1.4333	0.1233
PMeOH(18:1/26:2)	PMeOH	0.5564	0.0139	0.4309	0.0111	0.6570	0.0565
PG(18:1/18:2)	PG	0.6619	0.0165	0.6577	0.0169	0.7811	0.0672
TG(10:0/16:1/16:1)	TG	0.4164	0.0104	0.0673	0.0017	0.4905	0.0422
LNAPE(18:2/N/20:4)	LNAPE	1.7919	0.0448	1.9416	0.0499	2.1105	0.1816
PC(18:0/20:4)	PC	42.3416	1.0585	28.2687	0.7258	49.8684	4.2910
SHexCer(34:1;3O)	SHexCer	0.0539	0.0013	0.0711	0.0018	0.0634	0.0055
TG(15:0/16:0/16:1)	TG	1.4980	0.0375	0.4438	0.0114	1.7617	0.1516
PI(18:3;O/18:1)	PI	0.8972	0.0224	0.6687	0.0172	1.0512	0.0905
PS(18:0/18:1;3O)	PS	0.6937	0.0173	0.8344	0.0214	0.8090	0.0696
TG(15:1/17:0/17:0)	TG	9.1335	0.2283	1.8273	0.0469	10.6411	0.9156
Cer(13:2;2O/44:12)	Cer	104.5117	2.6128	66.9436	1.7188	121.6670	10.4690
PE/Cer(12:2;2O/13:1)	Cer	0.6883	0.0172	0.6958	0.0179	0.8008	0.0689
PC(34:5;O)	PC	0.0120	0.0003	0.0178	0.0005	0.0139	0.0012

TG(16:1/18:0/18:0)	TG	89.2956	2.2324	33.1131	0.8502	103.2945	8.8881
PE/Cer(12:2;2O/24:1;O)	Cer	34.8760	0.8719	74.8171	1.9210	40.2858	3.4665
LPE(20:1;O)	LPE	0.1729	0.0043	0.0756	0.0019	0.1997	0.0172
HexCer(16:3;2O/16:2;O)	HexCer	0.0240	0.0006	0.0411	0.0011	0.0276	0.0024
Cer(18:1;2O/19:0)	Cer	0.5372	0.0134	0.3680	0.0094	0.6177	0.0531
DG(16:0/22:4)	DG	8.2790	0.2070	4.8527	0.1246	9.5051	0.8179
PE/Cer(14:1;2O/26:0)	Cer	4.4344	0.1109	3.4657	0.0890	5.0783	0.4370
PG(22:4/22:4)	PG	0.3071	0.0077	0.5105	0.0131	0.3512	0.0302
LNAPE(18:1/N/26:7)	LNAPE	0.1535	0.0038	0.1630	0.0042	0.1753	0.0151
DG(16:1/18:2)	DG	4.6279	0.1157	0.9318	0.0239	5.2788	0.4542
PE(16:1;O/18:2)	PE	14.2532	0.3563	29.8332	0.7660	16.2419	1.3976
PG(18:0/18:2)	PG	1.2034	0.0301	0.6603	0.0170	1.3707	0.1179
PI(16:1;O/18:1)	PI	1.8444	0.0461	2.1469	0.0551	2.0932	0.1801
PE/Cer(18:1;2O/26:0)	Cer	2.3962	0.0599	2.0700	0.0531	2.6968	0.2321
PG(18:2/20:4)	PG	0.0861	0.0022	0.1571	0.0040	0.0969	0.0083
PI(11:0;O/24:1)	PI	0.0276	0.0007	0.0000	0.0000	0.0311	0.0027
TG(16:0/16:0/18:2)	TG	37.3058	0.9326	19.2971	0.4955	41.9206	3.6071
TG(17:1/19:0/19:0)	TG	1.3570	0.0339	0.6944	0.0178	1.5240	0.1311
PE(16:1;O/16:0)	PE	0.9421	0.0236	1.5980	0.0410	1.0567	0.0909
LNAPE(22:3/N/18:0)	LNAPE	0.4878	0.0122	0.8340	0.0214	0.5467	0.0470
PC(16:0;O)	PC	0.3833	0.0096	0.2095	0.0054	0.4262	0.0367
PI(18:1/20:4)	PI	1.3420	0.0336	2.0709	0.0532	1.4861	0.1279

TG(13:1/16:0/16:0)	TG	0.1412	0.0035	0.0551	0.0014	0.1562	0.0134
PI(16:0/22:4)	PI	1.3214	0.0330	3.0336	0.0779	1.4604	0.1257
PS(16:0/20:4;3O)	PS	0.1908	0.0048	0.2511	0.0064	0.2107	0.0181
TG(16:3/20:0/20:0)	TG	1.1094	0.0277	1.0444	0.0268	1.2237	0.1053
FA(24:1)	FA	3.1264	0.0782	1.5725	0.0404	3.4411	0.2961
PI(18:0/22:6)	PI	0.3592	0.0090	0.3128	0.0080	0.3950	0.0340
PI/Cer(12:2;2O/24:2;O)	Cer	2.0704	0.0518	2.4492	0.0629	2.2710	0.1954
TG(18:0/18:0/18:2)	TG	0.3624	0.0091	0.2416	0.0062	0.3972	0.0342
DG(18:1/20:2)	DG	1.1392	0.0285	0.5966	0.0153	1.2418	0.1069
PI(12:0;O/26:5)	PI	0.1059	0.0026	0.0599	0.0015	0.1155	0.0099
LNAPE(18:0/N/20:1)	LNAPE	1.0834	0.0271	1.2679	0.0326	1.1806	0.1016
PE(16:1;O/22:4)	PE	67.1392	1.6785	54.1663	1.3908	73.0332	6.2843
PC(16:1/20:3)	PC	6.6854	0.1671	6.3599	0.1633	7.2704	0.6256
FA(42:5)	FA	188.2325	4.7058	221.7684	5.6941	204.4920	17.5958
PE/Cer(16:3;2O/26:2;O)	Cer	5.4564	0.1364	4.7799	0.1227	5.9269	0.5100
PEtOH(18:2/22:4)	PEtOH	0.2697	0.0067	0.6824	0.0175	0.2912	0.0251
TG(16:0/16:1/16:1)	TG	58.9174	1.4729	29.5018	0.7575	63.5249	5.4661
PE(18:0/22:5)	PE	3.7550	0.0939	9.4376	0.2423	4.0381	0.3475
LNAPE(20:3/N/16:0)	LNAPE	4.0217	0.1005	9.3140	0.2391	4.3219	0.3719
LNAPE(18:2/N/16:0)	LNAPE	12.8455	0.3211	16.8372	0.4323	13.8032	1.1877
PC(20:0;O)	PC	0.0209	0.0005	0.0050	0.0001	0.0224	0.0019
PG(19:1;O/22:5)	PG	1.7210	0.0430	2.4414	0.0627	1.8471	0.1589

Cer(10:0;3O/30:1;(2OH))	Cer	6.6147	0.1654	5.2241	0.1341	7.0815	0.6093
PE(22:5;O/17:0)	PE	2.5968	0.0649	1.7336	0.0445	2.7792	0.2391
PI(20:5/22:6)	PI	0.0487	0.0012	0.0449	0.0012	0.0521	0.0045
DG(18:2/20:4)	DG	2.4004	0.0600	0.3277	0.0084	2.5566	0.2200
PC(35:2;O)	PC	0.2824	0.0071	0.3066	0.0079	0.3005	0.0259
PE(8:0;O/28:4)	PE	8.5905	0.2148	18.3015	0.4699	9.1298	0.7856
PE(16:2;O/22:6)	PE	0.0755	0.0019	0.1392	0.0036	0.0801	0.0069
PC(16:3;O/18:2)	PC	1.3052	0.0326	1.1389	0.0292	1.3820	0.1189
LPI(18:0)	LPI	1.3557	0.0339	0.7844	0.0201	1.4327	0.1233
PS(18:0/20:4)	PS	2.9237	0.0731	2.3418	0.0601	3.0875	0.2657
PG(18:0/18:0;O)	PG	0.6972	0.0174	1.7243	0.0443	0.7358	0.0633
HexCer(9:0;2O/30:5)	HexCer	0.2482	0.0062	1.1712	0.0301	0.2611	0.0225
Cer(10:0;3O/28:1;(2OH))	Cer	22.4807	0.5620	13.6105	0.3495	23.5472	2.0262
LPC(20:5)	LPC	1.4165	0.0354	0.4810	0.0124	1.4806	0.1274
PC(18:5;O/4:0)	PC	1.1309	0.0283	0.1963	0.0050	1.1793	0.1015
PS(10:0/24:1)	PS	1.1629	0.0291	0.8906	0.0229	1.2087	0.1040
PE(18:1/20:3)	PE	3.9259	0.0981	4.7055	0.1208	4.0699	0.3502
PE/Cer(12:1;2O/12:0;O)	Cer	1.2994	0.0325	1.2943	0.0332	1.3469	0.1159
Hex2Cer(38:1;2O)	Hex2Cer	0.2300	0.0057	0.2620	0.0067	0.2383	0.0205
PE(36:1)	PE	8.3223	0.2081	20.1537	0.5175	8.6025	0.7402
FA(44:5)	FA	47.2679	1.1817	52.1134	1.3380	48.6153	4.1832
PC(42:9)	PC	0.3202	0.0080	0.2317	0.0059	0.3292	0.0283

PC(35:6;O)	PC	22.3051	0.5576	29.0129	0.7449	22.9140	1.9717
PE(18:1;O/22:6)	PE	8.7481	0.2187	6.6705	0.1713	8.9685	0.7717
PC(16:0/20:4;2O)	PC	0.1713	0.0043	0.0000	0.0000	0.1755	0.0151
LPC(28:6)	LPC	0.2955	0.0074	0.4041	0.0104	0.3016	0.0259
PC(16:4;O/22:6)	PC	0.0964	0.0024	0.0959	0.0025	0.0984	0.0085
PE(17:1/17:1)	PE	29.2859	0.7321	36.9026	0.9475	29.7052	2.5560
PS(22:0/18:1)	PS	0.0461	0.0012	0.0475	0.0012	0.0467	0.0040
PS(18:0/18:1)	PS	1.4642	0.0366	1.2893	0.0331	1.4821	0.1275
LNAPE(18:1/N/18:0)	LNAPE	5.4631	0.1366	11.6521	0.2992	5.5070	0.4739
PE(16:0/16:1)	PE	0.2756	0.0069	0.6934	0.0178	0.2774	0.0239
PE/Cer(15:3;2O/26:2;O)	Cer	7.0592	0.1765	4.4789	0.1150	7.1038	0.6113
CerP(19:0;2O/2:0)	CerP	0.6489	0.0162	0.5405	0.0139	0.6493	0.0559
TG(16:0/18:1/18:2)	TG	431.9344	10.7984	178.7174	4.5887	432.1543	37.1854
PI(34:1)	PI	0.3081	0.0077	0.2554	0.0066	0.3069	0.0264
PC(36:4)	PC	70.2002	1.7550	79.1679	2.0327	69.8531	6.0106
TG(17:0/17:0/17:0)	TG	2.5157	0.0629	0.3053	0.0078	2.4922	0.2144
Cer(21:0;2O/15:1)	Cer	5.8595	0.1465	3.7037	0.0951	5.8014	0.4992
PC(18:1/22:4)	PC	11.5998	0.2900	6.4670	0.1660	11.4448	0.9848
LPE(22:5)	LPE	0.7132	0.0178	0.2478	0.0064	0.7028	0.0605
PC(31:0;O)	PC	0.7217	0.0180	0.4766	0.0122	0.7100	0.0611
PE(18:5;O/4:0)	PE	0.4606	0.0115	0.1593	0.0041	0.4462	0.0384
Cer(8:0;2O/28:1)	Cer	6.1985	0.1550	3.7812	0.0971	5.9998	0.5163

PE/Cer(21:2;2O/26:0;O)	Cer	3.8706	0.0968	2.7880	0.0716	3.7370	0.3216
PE(18:1;O/18:2)	PE	16.4493	0.4112	24.8652	0.6384	15.8445	1.3634
TG(15:0/16:0/16:0)	TG	1.0592	0.0265	0.1905	0.0049	1.0119	0.0871
PEtOH(19:1/24:4)	PEtOH	1.1574	0.0289	1.4618	0.0375	1.1043	0.0950
PE(18:2;O/22:5)	PE	7.5825	0.1896	9.7385	0.2500	7.2239	0.6216
PG(20:0/16:1)	PG	1.3488	0.0337	1.7684	0.0454	1.2781	0.1100
PE(16:1;O/20:4)	PE	52.4242	1.3106	61.0774	1.5682	49.3330	4.2449
HexCer(36:3;3O/15:1;(2OH))	HexCer	0.7600	0.0190	0.0639	0.0016	0.7152	0.0615
PC(33:3;O)	PC	4.5715	0.1143	6.8119	0.1749	4.2826	0.3685
TG(18:0/18:0/20:0)	TG	0.2710	0.0068	0.0379	0.0010	0.2530	0.0218
PC(16:0/20:3)	PC	40.0976	1.0024	51.6731	1.3267	37.3645	3.2151
TG(16:1/16:2/18:2)	TG	0.2602	0.0065	0.3198	0.0082	0.2403	0.0207
PS(12:0/26:4)	PS	14.4970	0.3624	6.8521	0.1759	13.3817	1.1515
TG(16:0/16:0/16:0)	TG	29.6278	0.7407	11.9757	0.3075	27.3390	2.3524
PI(2:0/7:0)	PI	0.0664	0.0017	0.1001	0.0026	0.0613	0.0053
HexCer(8:0;2O/32:7)	HexCer	0.6009	0.0150	0.7844	0.0201	0.5538	0.0476
PA(18:1/26:4)	PA	2.6021	0.0651	2.7426	0.0704	2.3957	0.2061
TG(16:0/18:0/18:1)	TG	233.0001	5.8250	46.3516	1.1901	214.3380	18.4430
LNAPE(18:1/N/22:0)	LNAPE	0.3332	0.0083	0.3859	0.0099	0.3063	0.0264
PC(31:2;O)	PC	0.0090	0.0002	0.1190	0.0031	0.0082	0.0007
PI(18:1/18:0;2O)	PI	0.0662	0.0017	0.0817	0.0021	0.0603	0.0052
PC(33:1)	PC	1.3027	0.0326	1.3765	0.0353	1.1826	0.1018

Cer(18:1;2O/24:2)	Cer	1.0675	0.0267	0.5661	0.0145	0.9667	0.0832
DG(16:0;O/22:6)	DG	0.1985	0.0050	0.0313	0.0008	0.1795	0.0154
HexCer(9:0;2O/40:11)	HexCer	0.1189	0.0030	0.2793	0.0072	0.1072	0.0092
TG(12:0/18:2/18:3)	TG	0.7804	0.0195	0.5289	0.0136	0.7037	0.0605
FA(24:6)	FA	18.1666	0.4542	4.0669	0.1044	16.3613	1.4078
PG(12:0;O/24:3)	PG	0.0112	0.0003	0.0066	0.0002	0.0101	0.0009
DG(14:1/18:2)	DG	0.1433	0.0036	0.0184	0.0005	0.1287	0.0111
PC(9:0;O/17:0)	PC	0.4581	0.0115	0.4767	0.0122	0.4110	0.0354
CAR(25:2)	CAR	0.0135	0.0003	0.0044	0.0001	0.0120	0.0010
PC(18:0/20:5)	PC	16.6147	0.4154	11.5507	0.2966	14.7744	1.2713
TG(16:0/18:0/24:1)	TG	1.2418	0.0310	0.1075	0.0028	1.1022	0.0948
Hex2Cer(42:1;2O)	Hex2Cer	0.1129	0.0028	0.1280	0.0033	0.0999	0.0086
HexCer(8:0;2O/34:9)	HexCer	0.3376	0.0084	0.2262	0.0058	0.2965	0.0255
PG(17:1;O/22:5)	PG	0.4399	0.0110	0.7062	0.0181	0.3835	0.0330
PC(17:0;O/20:4)	PC	0.0314	0.0008	0.0128	0.0003	0.0274	0.0024
LPE(20:5)	LPE	0.4113	0.0103	0.1407	0.0036	0.3573	0.0307
TG(16:0/18:1/24:1)	TG	4.4240	0.1106	0.3609	0.0093	3.8352	0.3300
PI(18:1/20:3;O)	PI	0.1383	0.0035	0.0064	0.0002	0.1195	0.0103
FA(24:2)	FA	3.9301	0.0983	1.0955	0.0281	3.3949	0.2921
PC(32:0)	PC	26.5312	0.6633	11.6763	0.2998	22.9155	1.9718
DGGA(16:1/16:1)	DGGA	0.5216	0.0130	0.4865	0.0125	0.4484	0.0386
Hex2Cer(17:0;2O/19:1)	Hex2Cer	0.1310	0.0033	0.3205	0.0082	0.1125	0.0097

Hex2Cer(42:2;2O)	Hex2Cer	0.0741	0.0019	0.1098	0.0028	0.0636	0.0055
PE(14:0/18:1)	PE	0.1621	0.0041	0.5419	0.0139	0.1388	0.0119
PC(22:4/22:4)	PC	0.0316	0.0008	0.0263	0.0007	0.0270	0.0023
PC(16:0/18:2)	PC	0.3898	0.0097	0.5636	0.0145	0.3306	0.0284
TG(16:0/16:0/17:0)	TG	3.5265	0.0882	0.5095	0.0131	2.9764	0.2561
HexCer(8:0;2O/34:8)	HexCer	3.6800	0.0920	5.2869	0.1357	3.1030	0.2670
Cer(8:0;2O/26:1)	Cer	6.6615	0.1665	2.5976	0.0667	5.6055	0.4823
PE/Cer(16:0;2O/26:1)	Cer	2.5302	0.0633	1.5544	0.0399	2.1249	0.1828
HexCer(8:0;2O/30:8)	HexCer	0.8211	0.0205	0.8891	0.0228	0.6879	0.0592
Cer(19:0;2O/15:1)	Cer	6.2999	0.1575	2.3483	0.0603	5.2509	0.4518
PE/Cer(15:0;2O/26:1)	Cer	0.2803	0.0070	0.1701	0.0044	0.2331	0.0201
PS(18:0/22:4)	PS	4.4447	0.1111	4.9183	0.1263	3.6922	0.3177
SHexCer(14:0;2O/38:1)	SHexCer	0.2392	0.0060	0.0051	0.0001	0.1980	0.0170
PC(16:1/18:1)	PC	237.0418	5.9260	220.3843	5.6585	195.6172	16.8322
Hex2Cer(36:1;2O)	Hex2Cer	0.0718	0.0018	0.1772	0.0046	0.0591	0.0051
TG(18:1/18:2/22:5)	TG	0.0715	0.0018	0.0270	0.0007	0.0588	0.0051
TG(18:0/18:0/18:1)	TG	60.9975	1.5249	10.8373	0.2783	49.8973	4.2935
TG(16:0/16:0/19:0)	TG	0.1067	0.0027	0.1209	0.0031	0.0873	0.0075
TG(15:0/16:1/16:1)	TG	1.2758	0.0319	0.3236	0.0083	1.0436	0.0898
TG(17:0/18:0/18:0)	TG	0.2848	0.0071	0.1854	0.0048	0.2321	0.0200
PC(33:0;O)	PC	0.1402	0.0035	0.2336	0.0060	0.1139	0.0098
PC(33:0)	PC	1.1407	0.0285	0.4151	0.0107	0.9192	0.0791

PMeOH(18:1/22:4)	PMeOH	0.0000	0.0000	0.0360	0.0009	0.0000	0.0000
PS(22:2/16:4)	PS	0.0000	0.0000	0.0253	0.0006	0.0000	0.0000
Cer(22:0;3O/24:0;(2OH))	Cer	0.0000	0.0000	0.0029	0.0001	0.0000	0.0000
HexCer(20:3;2O/13:1)	HexCer	0.0000	0.0000	0.0001	0.0000	0.0000	0.0000
PE/Cer(12:1;2O/18:0)	Cer	0.0000	0.0000	0.0000	0.0000	0.0000	0.0000
Cer(8:0;2O/39:0)	Cer	0.0000	0.0000	0.0000	0.0000	0.0000	0.0000
Cer(14:0;2O/35:0;(2OH))	Cer	0.0000	0.0000	0.0000	0.0000	0.0000	0.0000
HexCer(18:0;2O/22:0)	HexCer	0.0000	0.0000	0.0000	0.0000	0.0000	0.0000
LPC(38:7)	LPC	0.0000	0.0000	0.0000	0.0000	0.0000	0.0000
PC(12:0/44:11)	PC	0.0000	0.0000	0.0000	0.0000	0.0000	0.0000
PC(36:7)	PC	0.0000	0.0000	0.0000	0.0000	0.0000	0.0000
PEtOH(18:0/22:3)	PEtOH	0.0000	0.0000	0.0000	0.0000	0.0000	0.0000
PEtOH(18:1/24:1)	PEtOH	0.0000	0.0000	0.0000	0.0000	0.0000	0.0000
PMeOH(18:2/24:4)	PMeOH	0.0000	0.0000	0.0000	0.0000	0.0000	0.0000
TG(10:0/18:2/18:3)	TG	0.0000	0.0000	0.0000	0.0000	0.0000	0.0000
TG(16:0/18:0/22:5)	TG	0.0000	0.0000	0.0000	0.0000	0.0000	0.0000
LNAPE(20:4/N/18:1)	LNAPE	5.0229	0.1256	6.3705	0.1636	4.0259	0.3464
LNAPE(18:1/N/21:1)	LNAPE	0.0333	0.0008	0.0046	0.0001	0.0266	0.0023
Cer(14:0;2O/30:2;(2OH))	Cer	1.3163	0.0329	0.9282	0.0238	1.0481	0.0902
PE(18:1;O/20:4)	PE	1.2175	0.0304	0.9742	0.0250	0.9676	0.0833
LPC(22:6)	LPC	0.5741	0.0144	0.1228	0.0032	0.4532	0.0390
HBMP(12:0/16:4/16:3)	HBMP	0.1164	0.0029	0.2630	0.0068	0.0913	0.0079

PE(12:0;O/26:7)	PE	27.7334	0.6933	29.9322	0.7685	21.6641	1.8641
PC(18:2/22:5)	PC	0.8042	0.0201	0.5693	0.0146	0.6280	0.0540
PG(18:1/20:4)	PG	0.1313	0.0033	0.0723	0.0019	0.1024	0.0088
TG(18:1/18:1/24:1)	TG	1.9933	0.0498	0.2079	0.0053	1.5547	0.1338
PMeOH(26:2/22:4)	PMeOH	0.0800	0.0020	0.0990	0.0025	0.0624	0.0054
TG(18:0/18:1/20:3)	TG	1.2819	0.0320	0.5150	0.0132	0.9975	0.0858
PE/Cer(12:0;2O/26:1)	Cer	1.7160	0.0429	1.9769	0.0508	1.3271	0.1142
PC(17:2;O/20:3)	PC	0.5840	0.0146	0.7629	0.0196	0.4502	0.0387
TG(16:0/18:1/19:0)	TG	6.4576	0.1614	0.6868	0.0176	4.9720	0.4278
PE(18:0/22:4)	PE	9.1433	0.2286	13.0492	0.3350	7.0336	0.6052
TG(18:1/18:1/26:1)	TG	0.1065	0.0027	0.0132	0.0003	0.0815	0.0070
PC(38:3)	PC	0.2794	0.0070	0.0015	0.0000	0.2136	0.0184
PS(10:0/26:2)	PS	0.4529	0.0113	0.0254	0.0007	0.3459	0.0298
TG(14:0/16:1/18:2)	TG	33.8818	0.8470	17.4156	0.4472	25.8689	2.2259
HexCer(11:0;2O/44:12)	HexCer	0.0597	0.0015	0.0818	0.0021	0.0455	0.0039
PC(37:4)	PC	0.4500	0.0113	0.1456	0.0037	0.3395	0.0292
FA(24:4)	FA	6.4089	0.1602	1.0817	0.0278	4.8304	0.4156
TG(17:0/19:2/19:2)	TG	1.6858	0.0421	0.5571	0.0143	1.2633	0.1087
PC(37:6;O)	PC	1.5116	0.0378	1.5557	0.0399	1.1293	0.0972
PI(19:0/20:4)	PI	0.1306	0.0033	0.0862	0.0022	0.0976	0.0084
LNAPE(18:2/N/17:0)	LNAPE	0.5360	0.0134	0.2813	0.0072	0.3998	0.0344
DG(16:2/18:2)	DG	0.6821	0.0171	0.0276	0.0007	0.5084	0.0437

HexCer(16:0;2O/18:0;O)	HexCer	1.2462	0.0312	1.8003	0.0462	0.9286	0.0799
TG(13:1/21:2/21:2)	TG	0.9800	0.0245	0.4580	0.0118	0.7295	0.0628
PE(20:4/22:6)	PE	0.1899	0.0047	0.1436	0.0037	0.1412	0.0122
PC(17:0/17:1)	PC	0.7345	0.0184	2.2671	0.0582	0.5436	0.0468
TG(18:0/18:1/24:0)	TG	0.2842	0.0071	0.0335	0.0009	0.2094	0.0180
CAR(16:1)	CAR	0.0172	0.0004	0.0117	0.0003	0.0124	0.0011
TG(17:0/18:1/18:1)	TG	27.7478	0.6937	2.7563	0.0708	20.0250	1.7231
PS(18:1;O/22:6)	PS	0.4725	0.0118	1.0808	0.0277	0.3406	0.0293
TG(16:0/16:0/17:2)	TG	11.0172	0.2754	1.8173	0.0467	7.9201	0.6815
PC(42:8)	PC	0.3503	0.0088	0.2700	0.0069	0.2517	0.0217
TG(16:0/17:0/18:2)	TG	30.2894	0.7572	4.0608	0.1043	21.6921	1.8665
PI(18:0/20:4)	PI	62.0852	1.5521	72.8792	1.8712	44.4369	3.8236
PC(31:0)	PC	0.4071	0.0102	0.1309	0.0034	0.2914	0.0251
DG(18:2/22:5)	DG	0.7730	0.0193	0.0208	0.0005	0.5529	0.0476
LPS(18:0)	LPS	0.7950	0.0199	0.1153	0.0030	0.5672	0.0488
PE/Cer(12:2;2O/22:2;O)	Cer	0.3752	0.0094	1.1792	0.0303	0.2675	0.0230
TG(16:0/16:0/18:0)	TG	46.5702	1.1643	11.7680	0.3022	33.1798	2.8550
HexCer(21:1;2O/44:12)	HexCer	0.0138	0.0003	0.0121	0.0003	0.0098	0.0008
PC(33:6;O)	PC	4.1759	0.1044	5.2011	0.1335	2.9483	0.2537
PE(36:5)	PE	2.8298	0.0707	0.8969	0.0230	1.9970	0.1718
TG(17:1/19:1/19:1)	TG	0.1048	0.0026	0.0942	0.0024	0.0737	0.0063
LNAPE(18:1/N/16:0)	LNAPE	4.1004	0.1025	7.6543	0.1965	2.8562	0.2458

TG(18:1/18:1/24:0)	TG	0.9303	0.0233	0.1555	0.0040	0.6480	0.0558
Hex2Cer(29:0;2O)	Hex2Cer	0.0316	0.0008	0.0311	0.0008	0.0219	0.0019
PEtOH(18:1/22:6)	PEtOH	0.0546	0.0014	0.0873	0.0022	0.0372	0.0032
TG(16:1/16:1/18:1)	TG	19.7303	0.4933	6.7283	0.1728	13.4464	1.1570
SHexCer(15:3;2O/20:2;O)	SHexCer	0.0111	0.0003	0.0145	0.0004	0.0075	0.0006
DGGA(22:6/22:6)	DGGA	0.0296	0.0007	0.0000	0.0000	0.0200	0.0017
PE/Cer(13:1;2O/23:1;O)	Cer	34.0425	0.8511	80.9542	2.0786	22.9430	1.9742
HexCer(9:0;2O/42:11)	HexCer	0.3785	0.0095	0.2907	0.0075	0.2548	0.0219
PE(34:1)	PE	5.0494	0.1262	9.1811	0.2357	3.3898	0.2917
PC(18:5;O/16:0)	PC	4.1902	0.1048	2.3041	0.0592	2.8107	0.2419
PEtOH(19:0/20:4)	PEtOH	0.5644	0.0141	0.1410	0.0036	0.3785	0.0326
PC(33:2)	PC	1.1209	0.0280	0.7836	0.0201	0.7486	0.0644
TG(17:0/18:0/18:1)	TG	11.2746	0.2819	0.7028	0.0180	7.5134	0.6465
PE(16:1;O/18:1)	PE	13.4570	0.3364	31.7113	0.8142	8.9575	0.7708
TG(16:0/20:4/20:4)	TG	1.4142	0.0354	0.1674	0.0043	0.9409	0.0810
PC(17:0;O)	PC	0.0702	0.0018	0.0273	0.0007	0.0466	0.0040
PMeOH(22:4/22:4)	PMeOH	0.0774	0.0019	0.1445	0.0037	0.0513	0.0044
PC(40:7)	PC	0.4162	0.0104	0.5140	0.0132	0.2754	0.0237
PE(20:3/20:3)	PE	21.9264	0.5482	19.5715	0.5025	14.4865	1.2465
PC(22:0/18:1)	PC	0.5147	0.0129	0.4285	0.0110	0.3392	0.0292
PS(14:1/26:4)	PS	3.7002	0.0925	2.0606	0.0529	2.4249	0.2087
TG(14:0/14:0/18:1)	TG	10.0182	0.2505	2.2217	0.0570	6.5434	0.5630

PE(38:7)	PE	0.1939	0.0048	0.0974	0.0025	0.1260	0.0108
Cer(29:0;2O/19:0;(2OH))	Cer	0.0014	0.0000	0.0013	0.0000	0.0009	0.0001
PC(32:3;O)	PC	0.0328	0.0008	0.0396	0.0010	0.0211	0.0018
TG(9:0/16:0/18:1)	TG	0.1011	0.0025	0.0141	0.0004	0.0645	0.0056
PI(16:0/20:4)	PI	1.3550	0.0339	1.0004	0.0257	0.8633	0.0743
TG(14:0/16:1/16:1)	TG	0.5748	0.0144	0.2467	0.0063	0.3644	0.0314
TG(18:0/18:1/18:1)	TG	73.0901	1.8273	24.6444	0.6328	46.0560	3.9630
Cer(18:1;2O/26:0)	Cer	0.0406	0.0010	0.0446	0.0011	0.0255	0.0022
PE(16:3;O/22:5)	PE	0.0354	0.0009	0.0414	0.0011	0.0222	0.0019
TG(16:0/18:2/18:2)	TG	343.0578	8.5764	85.8434	2.2041	214.7789	18.4810
DG(18:2/18:2)	DG	18.5859	0.4646	0.8348	0.0214	11.5822	0.9966
DGGA(18:4/20:4)	DGGA	0.0927	0.0023	0.0718	0.0018	0.0577	0.0050
HexCer(16:1;2O/44:12)	HexCer	0.1323	0.0033	0.0023	0.0001	0.0824	0.0071
PE(18:2;O/22:6)	PE	5.2392	0.1310	6.1547	0.1580	3.2606	0.2806
PI(17:0/17:1)	PI	0.9704	0.0243	0.2331	0.0060	0.6037	0.0519
HexCer(14:0;2O/44:12)	HexCer	0.4107	0.0103	0.1341	0.0034	0.2538	0.0218
TG(16:1/18:0/22:0)	TG	6.7712	0.1693	0.4289	0.0110	4.1801	0.3597
PC(40:5)	PC	2.2612	0.0565	8.1259	0.2086	1.3949	0.1200
TG(14:0/16:0/16:0)	TG	1.1490	0.0287	0.2327	0.0060	0.7035	0.0605
PC(18:1/22:6)	PC	0.8714	0.0218	1.8866	0.0484	0.5328	0.0458
TG(16:0/18:1/23:0)	TG	0.4178	0.0104	0.0346	0.0009	0.2552	0.0220
PE(18:0/20:4;4O)	PE	0.1775	0.0044	0.0000	0.0000	0.1083	0.0093

PC(20:4/20:4)	PC	2.8026	0.0701	3.1921	0.0820	1.7098	0.1471
TG(18:2/18:2/24:1)	TG	0.6267	0.0157	0.0591	0.0015	0.3812	0.0328
LPC(22:5)	LPC	0.9547	0.0239	0.0353	0.0009	0.5799	0.0499
TG(12:0/22:2/22:2)	TG	23.0526	0.5763	3.5765	0.0918	13.9669	1.2018
LNAPE(18:1/N/17:0)	LNAPE	0.0667	0.0017	0.0707	0.0018	0.0403	0.0035
TG(18:0/18:0/19:0)	TG	0.3796	0.0095	0.0815	0.0021	0.2296	0.0198
TG(18:1/18:1/22:1)	TG	7.3539	0.1838	0.4988	0.0128	4.4467	0.3826
PS(16:0/18:2)	PS	0.8812	0.0220	0.6004	0.0154	0.5310	0.0457
TG(14:0/18:2/18:2)	TG	85.5583	2.1390	27.6542	0.7100	51.3552	4.4189
PE(16:0;O/20:1)	PE	2.1267	0.0532	2.9518	0.0758	1.2762	0.1098
PE(17:1;O/20:4)	PE	1.1678	0.0292	0.4186	0.0107	0.6999	0.0602
PC(16:4/30:8)	PC	1.5383	0.0385	1.6487	0.0423	0.9203	0.0792
LNAPE(18:2/N/22:5)	LNAPE	0.1270	0.0032	0.6639	0.0170	0.0760	0.0065
PI(10:0;O/26:5)	PI	0.3412	0.0085	0.2232	0.0057	0.2031	0.0175
DG(16:1/16:1)	DG	21.3119	0.5328	5.0564	0.1298	12.6383	1.0875
PC(17:0/20:4)	PC	0.5260	0.0131	0.2063	0.0053	0.3088	0.0266
Hex2Cer(30:1;2O)	Hex2Cer	0.0010	0.0000	0.0002	0.0000	0.0006	0.0000
Cer(14:0;2O/34:1;(2OH))	Cer	0.0013	0.0000	0.0007	0.0000	0.0007	0.0001
PE(16:3/26:7)	PE	0.1420	0.0036	0.0961	0.0025	0.0819	0.0070
TG(18:0/18:1/22:0)	TG	0.4098	0.0102	0.0318	0.0008	0.2347	0.0202
PC(18:0;O/20:0)	PC	0.1365	0.0034	0.0699	0.0018	0.0776	0.0067
PC(37:6)	PC	0.0471	0.0012	0.0465	0.0012	0.0268	0.0023

HexCer(8:0;2O/28:6)	HexCer	0.0084	0.0002	0.0195	0.0005	0.0048	0.0004
HexCer(9:0;2O/30:8)	HexCer	0.5341	0.0134	0.1133	0.0029	0.3015	0.0259
TG(17:1/17:1/17:2)	TG	0.7775	0.0194	0.1085	0.0028	0.4386	0.0377
TG(9:0/18:1/18:1)	TG	0.2396	0.0060	0.0571	0.0015	0.1351	0.0116
LNAPE(18:2/N/20:5)	LNAPE	0.1501	0.0038	0.0781	0.0020	0.0844	0.0073
PC(14:1/14:1)	PC	0.0078	0.0002	0.0139	0.0004	0.0043	0.0004
Hex2Cer(17:0;2O/17:1)	Hex2Cer	0.1276	0.0032	0.0895	0.0023	0.0703	0.0060
PEtOH(16:0/18:1)	PEtOH	0.0396	0.0010	0.0457	0.0012	0.0218	0.0019
TG(13:1/18:0/18:0)	TG	0.1021	0.0026	0.0333	0.0009	0.0559	0.0048
TG(18:1/18:2/24:1)	TG	1.5951	0.0399	0.1156	0.0030	0.8730	0.0751
SHexCer(14:1;2O/23:1)	SHexCer	0.2363	0.0059	0.2339	0.0060	0.1293	0.0111
PG(18:0/20:4)	PG	0.6878	0.0172	0.2102	0.0054	0.3757	0.0323
DG(9:0;O/28:6)	DG	0.2492	0.0062	0.1608	0.0041	0.1357	0.0117
PE(38:5)	PE	1.4785	0.0370	1.6880	0.0433	0.7998	0.0688
LPE(22:6)	LPE	0.7300	0.0182	0.3041	0.0078	0.3944	0.0339
TG(17:0/17:0/25:0)	TG	0.0136	0.0003	0.0019	0.0000	0.0074	0.0006
TG(16:0/18:1/18:1)	TG	0.0298	0.0007	0.0218	0.0006	0.0158	0.0014
TG(15:1/21:2/21:2)	TG	1.0165	0.0254	0.4519	0.0116	0.5371	0.0462
LNAPE(18:2/N/16:1)	LNAPE	0.6213	0.0155	0.4821	0.0124	0.3282	0.0282
TG(18:1/18:1/20:1)	TG	33.5667	0.8392	4.3743	0.1123	17.6316	1.5171
HexCer(18:1;2O/20:0)	HexCer	0.0970	0.0024	0.0391	0.0010	0.0508	0.0044
PEtOH(18:3/24:4)	PEtOH	1.3917	0.0348	1.1673	0.0300	0.7126	0.0613

HBMP(12:0/12:0/16:3)	HBMP	0.1158	0.0029	0.1452	0.0037	0.0591	0.0051
LNAPE(18:3/N/16:0)	LNAPE	0.1898	0.0047	0.1347	0.0035	0.0968	0.0083
PE/Cer(16:0;2O/26:2)	Cer	2.3631	0.0591	1.2316	0.0316	1.2036	0.1036
TG(18:0/18:1/19:0)	TG	2.1078	0.0527	0.1096	0.0028	1.0724	0.0923
LPE(17:0)	LPE	0.3741	0.0094	0.0680	0.0017	0.1902	0.0164
TG(13:1/18:1/18:1)	TG	5.7398	0.1435	0.9596	0.0246	2.9156	0.2509
SHexCer(14:0;2O/22:1)	SHexCer	0.0955	0.0024	0.0636	0.0016	0.0485	0.0042
PE(17:1;O/22:6)	PE	0.4350	0.0109	0.1679	0.0043	0.2204	0.0190
TG(9:0/16:0/18:2)	TG	0.1779	0.0044	0.0115	0.0003	0.0896	0.0077
Cer(18:1;3O/26:0;(2OH))	Cer	0.0030	0.0001	0.0020	0.0001	0.0015	0.0001
PC(17:1;O/18:1)	PC	0.3571	0.0089	0.2408	0.0062	0.1791	0.0154
TG(14:1/20:0/20:0)	TG	0.2972	0.0074	0.0843	0.0022	0.1482	0.0128
CAR(18:1)	CAR	0.3261	0.0082	0.1404	0.0036	0.1626	0.0140
TG(14:0/14:0/14:0)	TG	0.2611	0.0065	0.0232	0.0006	0.1298	0.0112
TG(18:0/18:1/21:0)	TG	0.2838	0.0071	0.0192	0.0005	0.1410	0.0121
TG(14:0/14:0/16:1)	TG	1.3245	0.0331	0.2235	0.0057	0.6567	0.0565
PC(39:5)	PC	0.2494	0.0062	0.0583	0.0015	0.1219	0.0105
TG(16:1/16:1/16:1)	TG	1.7617	0.0440	0.6616	0.0170	0.8483	0.0730
Cer(18:0;2O/24:0)	Cer	0.1272	0.0032	0.0689	0.0018	0.0612	0.0053
TG(13:1/18:1/18:2)	TG	1.5128	0.0378	0.4031	0.0104	0.7122	0.0613
BMP(18:2/18:2)	BMP	8.4190	0.2105	7.2906	0.1872	3.8993	0.3355
PE/Cer(15:3;2O/22:2;O)	Cer	0.0544	0.0014	0.0000	0.0000	0.0252	0.0022

Cer(16:0;2O/19:1)	Cer	0.0470	0.0012	0.0090	0.0002	0.0215	0.0019
PI(13:1/24:6)	PI	0.0717	0.0018	0.0632	0.0016	0.0328	0.0028
Hex2Cer(34:1;2O)	Hex2Cer	0.0658	0.0016	0.0333	0.0009	0.0301	0.0026
TG(12:0/14:0/18:1)	TG	0.1590	0.0040	0.0407	0.0010	0.0727	0.0063
PA(20:4/24:4)	PA	0.3187	0.0080	0.0276	0.0007	0.1452	0.0125
TG(18:1/18:1/26:0)	TG	0.1077	0.0027	0.0152	0.0004	0.0490	0.0042
DG(18:2/22:6)	DG	1.0108	0.0253	0.1386	0.0036	0.4540	0.0391
HexCer(23:1;2O/44:12)	HexCer	0.0145	0.0004	0.0105	0.0003	0.0065	0.0006
PC(17:1/18:1)	PC	2.3185	0.0580	0.8898	0.0228	1.0389	0.0894
TG(16:0/18:0/18:0)	TG	29.8320	0.7458	3.0465	0.0782	13.3167	1.1459
PE(20:0/24:0)	PE	0.5254	0.0131	0.3172	0.0081	0.2342	0.0202
TG(15:0/18:1/18:2)	TG	24.5902	0.6148	3.0193	0.0775	10.9418	0.9415
TG(14:1/16:0/16:0)	TG	1.5056	0.0376	0.3952	0.0101	0.6699	0.0576
PE(18:2/34:2)	PE	0.8014	0.0200	0.0092	0.0002	0.3538	0.0304
PC(18:1;O/18:1)	PC	0.2762	0.0069	0.1353	0.0035	0.1215	0.0105
TG(16:1/16:1/20:3)	TG	0.3785	0.0095	0.0591	0.0015	0.1655	0.0142
TG(18:2/18:2/26:1)	TG	0.1540	0.0039	0.0108	0.0003	0.0667	0.0057
SHexCer(41:1;3O)	SHexCer	0.0039	0.0001	0.0000	0.0000	0.0017	0.0001
HexCer(18:1;2O/18:0)	HexCer	0.1499	0.0037	0.0822	0.0021	0.0648	0.0056
HexCer(8:1;2O/34:9)	HexCer	11.3543	0.2839	11.5370	0.2962	4.9036	0.4219
TG(12:0/16:0/18:2)	TG	10.2129	0.2553	2.6473	0.0680	4.3956	0.3782
PE(32:1/20:4)	PE	2.4247	0.0606	0.0687	0.0018	1.0420	0.0897

TG(18:1/18:1/25:0)	TG	0.0514	0.0013	0.0049	0.0001	0.0219	0.0019
PC(14:1;O/26:7)	PC	0.1264	0.0032	0.0042	0.0001	0.0536	0.0046
PC(29:2)	PC	0.0161	0.0004	0.0001	0.0000	0.0068	0.0006
TG(18:1/18:1/23:0)	TG	0.1464	0.0037	0.0089	0.0002	0.0614	0.0053
TG(16:3/16:3/18:0)	TG	1.6955	0.0424	0.3275	0.0084	0.7076	0.0609
PC(37:3)	PC	0.1650	0.0041	0.0947	0.0024	0.0688	0.0059
PS(18:0/22:5)	PS	5.8545	0.1464	1.8646	0.0479	2.4161	0.2079
TG(18:1/18:2/23:0)	TG	0.5136	0.0128	0.0333	0.0009	0.2116	0.0182
PC(20:0;O/28:0)	PC	1.4663	0.0367	0.1722	0.0044	0.6037	0.0519
LNAPE(18:1/N/26:0)	LNAPE	1.9928	0.0498	0.7970	0.0205	0.8186	0.0704
TG(18:1/18:1/18:2)	TG	341.4526	8.5363	80.2218	2.0597	139.4011	11.9950
PG(22:0;O/22:6)	PG	0.0689	0.0017	0.0565	0.0015	0.0279	0.0024
CAR(20:2)	CAR	0.1902	0.0048	0.0595	0.0015	0.0770	0.0066
PE(19:1/19:1)	PE	14.2704	0.3568	4.7297	0.1214	5.7473	0.4945
HexCer(16:1;2O/24:1;O)	HexCer	0.8388	0.0210	0.3403	0.0087	0.3373	0.0290
PEtOH(22:3/20:4)	PEtOH	0.0273	0.0007	0.0325	0.0008	0.0109	0.0009
PE(18:1;O/18:1)	PE	12.5562	0.3139	13.9995	0.3594	4.9715	0.4278
Cer(8:0;2O/33:0)	Cer	0.0285	0.0007	0.0103	0.0003	0.0113	0.0010
TG(15:1/16:1/16:1)	TG	0.9117	0.0228	0.1725	0.0044	0.3601	0.0310
DGGA(18:1/18:1)	DGGA	0.9322	0.0233	0.2784	0.0071	0.3673	0.0316
TG(18:0/18:1/23:0)	TG	0.0930	0.0023	0.0054	0.0001	0.0366	0.0031
SMGDG(17:0;O/28:6)	SMGDG	0.0219	0.0005	0.0182	0.0005	0.0085	0.0007

PE(17:1;O/18:1)	PE	0.4170	0.0104	0.6312	0.0162	0.1621	0.0140
TG(18:1/18:2/20:4)	TG	0.8967	0.0224	0.2877	0.0074	0.3476	0.0299
HexCer(9:0;2O/32:9)	HexCer	0.0281	0.0007	0.0312	0.0008	0.0109	0.0009
HexCer(18:1;2O/22:0)	HexCer	0.2302	0.0058	0.0205	0.0005	0.0887	0.0076
TG(18:1/18:2/20:2)	TG	5.8550	0.1464	0.6630	0.0170	2.2034	0.1896
PEtOH(21:1/22:5)	PEtOH	1.4742	0.0369	0.5752	0.0148	0.5532	0.0476
PE(11:0;O/28:7)	PE	0.1720	0.0043	0.0398	0.0010	0.0644	0.0055
PE(16:1;O/20:5)	PE	12.2703	0.3068	5.2618	0.1351	4.5318	0.3899
TG(11:0/18:2/18:2)	TG	0.4599	0.0115	0.1125	0.0029	0.1696	0.0146
TG(16:0/18:0/22:6)	TG	0.0433	0.0011	0.0000	0.0000	0.0159	0.0014
HexCer(16:1;2O/24:0;O)	HexCer	1.0033	0.0251	0.5995	0.0154	0.3675	0.0316
LNAPE(18:1/N/19:0)	LNAPE	0.0371	0.0009	0.0540	0.0014	0.0136	0.0012
HexCer(8:1;2O/32:9)	HexCer	1.1155	0.0279	1.0894	0.0280	0.4036	0.0347
PC(30:1)	PC	0.1627	0.0041	0.0847	0.0022	0.0588	0.0051
PC(17:2/17:2)	PC	0.3801	0.0095	0.1654	0.0042	0.1360	0.0117
PC(15:0/16:0)	PC	0.5615	0.0140	0.1260	0.0032	0.2005	0.0173
TG(18:2/18:2/22:0)	TG	4.6385	0.1160	0.3265	0.0084	1.6440	0.1415
PI(16:0/22:6)	PI	0.1134	0.0028	0.0943	0.0024	0.0396	0.0034
PE(18:0;O/20:1)	PE	1.2706	0.0318	0.8557	0.0220	0.4248	0.0365
PE/Cer(17:2;2O/26:0;O)	Cer	0.1913	0.0048	0.0000	0.0000	0.0639	0.0055
PC(16:3/26:7)	PC	0.1500	0.0038	0.0887	0.0023	0.0500	0.0043
TG(18:1/18:2/26:1)	TG	0.2854	0.0071	0.0267	0.0007	0.0934	0.0080

PE(22:6/22:6)	PE	0.0597	0.0015	0.0751	0.0019	0.0194	0.0017
PE(21:1)	PE	0.1164	0.0029	0.1053	0.0027	0.0375	0.0032
PE/Cer(22:0;2O/26:6)	Cer	31.8405	0.7960	18.3192	0.4704	10.2260	0.8799
TG(10:0/18:2/18:2)	TG	0.9594	0.0240	0.0000	0.0000	0.3076	0.0265
TG(12:0/18:2/18:2)	TG	14.5187	0.3630	4.8329	0.1241	4.6419	0.3994
PC(22:5/22:5)	PC	0.1420	0.0036	0.1335	0.0034	0.0448	0.0039
PC(16:4;O/20:4)	PC	0.2946	0.0074	0.1840	0.0047	0.0925	0.0080
DG(18:2/18:3)	DG	3.1327	0.0783	0.0772	0.0020	0.9811	0.0844
LNAPE(16:0/N/22:6)	LNAPE	4.1428	0.1036	2.2189	0.0570	1.2966	0.1116
PC(22:0;O/28:1)	PC	0.8717	0.0218	0.1479	0.0038	0.2725	0.0235
PC(32:3)	PC	0.0689	0.0017	0.0423	0.0011	0.0215	0.0019
SHexCer(14:0;2O/38:2)	SHexCer	0.5307	0.0133	0.0064	0.0002	0.1652	0.0142
CAR(20:1)	CAR	0.4381	0.0110	0.0311	0.0008	0.1359	0.0117
PC(20:0;O/18:2)	PC	1.0662	0.0267	0.7196	0.0185	0.3305	0.0284
LNAPE(16:0/N/24:4)	LNAPE	0.6161	0.0154	0.3010	0.0077	0.1908	0.0164
PC(16:0/18:2;2O)	PC	0.3956	0.0099	0.0090	0.0002	0.1219	0.0105
PE(40:5)	PE	0.3263	0.0082	0.0925	0.0024	0.1001	0.0086
PC(19:1/19:1)	PC	0.0209	0.0005	0.0027	0.0001	0.0064	0.0006
TG(18:1/18:2/21:0)	TG	1.3124	0.0328	0.0530	0.0014	0.3986	0.0343
TG(18:0/18:0/18:0)	TG	5.7510	0.1438	0.4112	0.0106	1.7266	0.1486
CAR(20:0)	CAR	1.1529	0.0288	0.1431	0.0037	0.3443	0.0296
TG(8:0/18:1/18:2)	TG	0.7883	0.0197	0.1619	0.0042	0.2348	0.0202

LNAPE(18:2/N/22:4)	LNAPE	0.0346	0.0009	0.0288	0.0007	0.0102	0.0009
TG(18:1/18:2/25:0)	TG	0.1158	0.0029	0.0052	0.0001	0.0337	0.0029
HexCer(8:0;2O/38:8)	HexCer	0.0098	0.0002	0.0054	0.0001	0.0028	0.0002
PC(28:4;O/18:1)	PC	1.1129	0.0278	0.0000	0.0000	0.3225	0.0277
PE/Cer(23:1;2O/26:1;O)	Cer	2.5528	0.0638	1.1244	0.0289	0.7316	0.0630
Cer(14:0;2O/14:0;O(FA 17:0))	Cer	2.4808	0.0620	0.9477	0.0243	0.6944	0.0598
PMeOH(18:0/18:2)	PMeOH	0.4454	0.0111	0.0139	0.0004	0.1235	0.0106
PS(10:0/24:2)	PS	0.3735	0.0093	0.3647	0.0094	0.1034	0.0089
PC(13:0;O/28:5)	PC	0.0621	0.0016	0.0449	0.0012	0.0171	0.0015
PI(16:2/22:4)	PI	0.0650	0.0016	0.0488	0.0013	0.0177	0.0015
PC(17:1;O/18:2)	PC	0.7259	0.0181	0.3020	0.0078	0.1964	0.0169
Hex2Cer(31:1;2O)	Hex2Cer	0.0235	0.0006	0.0199	0.0005	0.0063	0.0005
PC(20:4;O/28:7)	PC	0.0033	0.0001	0.0054	0.0001	0.0009	0.0001
PC(18:2/24:5)	PC	0.1453	0.0036	0.1093	0.0028	0.0390	0.0034
LPC(34:2)	LPC	0.1376	0.0034	0.0286	0.0007	0.0367	0.0032
Cer(18:1;2O/22:0;(2OH))	Cer	0.0712	0.0018	0.0333	0.0009	0.0188	0.0016
TG(16:1/18:2/18:2)	TG	193.8546	4.8464	20.2940	0.5211	49.8015	4.2852
TG(18:1/18:2/20:3)	TG	4.6908	0.1173	0.4342	0.0111	1.2007	0.1033
LPC(22:0)	LPC	0.0614	0.0015	0.0116	0.0003	0.0157	0.0013
TG(12:0/16:0/16:0)	TG	1.2078	0.0302	0.1145	0.0029	0.3054	0.0263
DG(18:1/40:9)	DG	6.1909	0.1548	0.1032	0.0026	1.5313	0.1318
PI(5:0/18:4)	PI	0.0078	0.0002	0.0215	0.0006	0.0019	0.0002

TG(16:0/20:4/22:6)	TG	0.7548	0.0189	0.0561	0.0014	0.1847	0.0159
PC(20:4/22:6)	PC	0.2190	0.0055	0.1278	0.0033	0.0534	0.0046
TG(16:3/20:1/20:1)	TG	0.4722	0.0118	0.0522	0.0013	0.1143	0.0098
PC(24:0/18:1)	PC	0.3367	0.0084	0.2743	0.0070	0.0795	0.0068
PS(18:0/20:4;3O)	PS	0.9641	0.0241	0.7525	0.0193	0.2259	0.0194
TG(16:1/18:2/18:3)	TG	0.5908	0.0148	0.0706	0.0018	0.1365	0.0117
LNAPE(16:0/N/26:1)	LNAPE	0.8115	0.0203	0.4494	0.0115	0.1859	0.0160
TG(16:0/16:0/24:0)	TG	0.8715	0.0218	0.0965	0.0025	0.1996	0.0172
PG(3:0/20:4)	PG	0.0428	0.0011	0.0338	0.0009	0.0098	0.0008
PE(18:1;O/20:1)	PE	7.4414	0.1860	5.8779	0.1509	1.6573	0.1426
TG(16:0/18:0/26:0)	TG	0.0967	0.0024	0.0197	0.0005	0.0214	0.0018
LNAPE(17:0/N/20:5)	LNAPE	0.0979	0.0024	0.0017	0.0000	0.0216	0.0019
PEtOH(18:0/25:0)	PEtOH	0.8674	0.0217	0.4413	0.0113	0.1913	0.0165
PC(34:5)	PC	0.0051	0.0001	0.0065	0.0002	0.0011	0.0001
PE/Cer(14:1;2O/26:0;O)	Cer	0.7435	0.0186	0.6942	0.0178	0.1611	0.0139
TG(15:1/18:1/18:2)	TG	11.3222	0.2831	0.7181	0.0184	2.4353	0.2095
PC(37:5)	PC	0.1940	0.0048	0.0213	0.0005	0.0415	0.0036
HBMP(12:0/12:0/12:0)	HBMP	0.0060	0.0001	0.0105	0.0003	0.0013	0.0001
TG(16:1/20:4/20:4)	TG	5.0342	0.1259	0.2101	0.0054	1.0512	0.0905
SHexCer(14:0;2O/28:2)	SHexCer	1.7336	0.0433	1.5801	0.0406	0.3583	0.0308
PE/Cer(13:0;2O/25:1;O)	Cer	1.2818	0.0320	1.9484	0.0500	0.2613	0.0225
SHexCer(14:0;2O/34:0)	SHexCer	2.7840	0.0696	0.0121	0.0003	0.5655	0.0487

PG(23:0;O/26:6)	PG	0.0297	0.0007	0.0390	0.0010	0.0059	0.0005
CAR(24:0)	CAR	0.0030	0.0001	0.0000	0.0000	0.0006	0.0001
HexCer(17:0;2O/25:1;O)	HexCer	0.4271	0.0107	0.3154	0.0081	0.0836	0.0072
TG(17:0/18:2/18:2)	TG	22.2408	0.5560	1.3023	0.0334	4.3310	0.3727
PI(17:0/20:4)	PI	0.2542	0.0064	0.1165	0.0030	0.0495	0.0043
TG(18:1/18:2/19:1)	TG	7.8730	0.1968	0.3717	0.0095	1.5222	0.1310
SHexCer(14:0;2O/27:1)	SHexCer	0.0128	0.0003	0.0069	0.0002	0.0024	0.0002
TG(16:1;O/20:0/20:0)	TG	0.5911	0.0148	0.0049	0.0001	0.1123	0.0097
PC(14:0/20:4)	PC	1.5259	0.0381	0.1424	0.0037	0.2851	0.0245
Cer(18:1;2O/24:1;(2OH))	Cer	0.0459	0.0011	0.0206	0.0005	0.0086	0.0007
Cer(27:1;2O/17:0;(2OH))	Cer	0.0065	0.0002	0.0024	0.0001	0.0012	0.0001
LPE(18:3;O)	LPE	0.2708	0.0068	0.0315	0.0008	0.0479	0.0041
TG(15:1/18:2/18:2)	TG	2.9412	0.0735	0.1170	0.0030	0.5153	0.0443
TG(16:0/18:3/18:3)	TG	32.2840	0.8071	1.9479	0.0500	5.5221	0.4752
SHexCer(14:0;2O/28:1)	SHexCer	0.1751	0.0044	0.1452	0.0037	0.0294	0.0025
CAR(22:0)	CAR	0.4161	0.0104	0.0585	0.0015	0.0698	0.0060
TG(16:0;O/20:1/20:1)	TG	2.3595	0.0590	0.0230	0.0006	0.3811	0.0328
TG(18:2/18:2/23:0)	TG	0.3349	0.0084	0.0045	0.0001	0.0539	0.0046
LNAPE(18:2/N/24:4)	LNAPE	0.2343	0.0059	0.0569	0.0015	0.0376	0.0032
PMeOH(16:0/18:2)	PMeOH	0.9631	0.0241	0.0445	0.0011	0.1517	0.0131
HexCer(16:1;2O/26:0;O)	HexCer	1.1601	0.0290	0.7231	0.0186	0.1808	0.0156
LNAPE(17:0/N/22:5)	LNAPE	0.2888	0.0072	0.0000	0.0000	0.0449	0.0039

PE(16:2/36:4)	PE	1.0244	0.0256	0.0037	0.0001	0.1570	0.0135
HexCer(16:0;2O/24:0;O)	HexCer	0.1707	0.0043	0.0850	0.0022	0.0260	0.0022
HexCer(8:0;2O/34:1)	HexCer	0.1715	0.0043	0.1030	0.0026	0.0261	0.0022
PC(16:0/26:6)	PC	0.0715	0.0018	0.0219	0.0006	0.0107	0.0009
TG(14:1/16:1/16:1)	TG	0.1577	0.0039	0.0377	0.0010	0.0235	0.0020
Cer(18:1;2O/24:0;(2OH))	Cer	0.1191	0.0030	0.0580	0.0015	0.0172	0.0015
TG(18:2/18:2/19:2)	TG	0.7911	0.0198	0.0317	0.0008	0.1139	0.0098
CAR(22:1)	CAR	0.7065	0.0177	0.0187	0.0005	0.1012	0.0087
PE(O/18:1;O/22:1)	PE	0.8792	0.0220	0.6625	0.0170	0.1229	0.0106
PEtOH(18:0/26:4)	PEtOH	0.3218	0.0080	0.2806	0.0072	0.0448	0.0039
PC(38:7)	PC	0.1089	0.0027	0.1670	0.0043	0.0151	0.0013
TG(18:1/18:2/18:2)	TG	63.3311	1.5833	3.0772	0.0790	8.4749	0.7292
Cer(29:0;2O/15:0;(2OH))	Cer	0.0128	0.0003	0.0025	0.0001	0.0017	0.0001
HexCer(18:1;2O/24:0)	HexCer	0.4554	0.0114	0.2682	0.0069	0.0574	0.0049
PC(20:2/20:2)	PC	0.4123	0.0103	0.0350	0.0009	0.0518	0.0045
TG(18:2/18:2/19:1)	TG	3.2092	0.0802	0.1430	0.0037	0.4026	0.0346
Cer(8:0;2O/41:0)	Cer	0.0001	0.0000	0.0002	0.0000	0.0000	0.0000
TG(17:1/18:2/18:2)	TG	8.2785	0.2070	0.2391	0.0061	1.0341	0.0890
Cer(11:0;2O/44:11)	Cer	1.2084	0.0302	0.7149	0.0184	0.1507	0.0130
PC(16:4;O/18:1)	PC	0.2128	0.0053	0.0715	0.0018	0.0254	0.0022
Cer(15:0;2O/16:5;(3OH)(FA 20:4))	Cer	0.0893	0.0022	0.0973	0.0025	0.0104	0.0009
PC(18:1/24:1)	PC	0.5795	0.0145	0.3009	0.0077	0.0669	0.0058

PI(18:4;O/26:7)	PI	0.0088	0.0002	0.0000	0.0000	0.0010	0.0001
PI(8:0/24:1)	PI	0.0528	0.0013	0.0323	0.0008	0.0060	0.0005
PMeOH(18:0/26:2)	PMeOH	0.0499	0.0012	0.0000	0.0000	0.0056	0.0005
HexCer(8:0;2O/30:4)	HexCer	0.4286	0.0107	0.3190	0.0082	0.0479	0.0041
PE/Cer(22:0;2O/26:5)	Cer	5.7343	0.1434	2.7551	0.0707	0.6298	0.0542
HexCer(18:1;2O/26:1)	HexCer	0.0284	0.0007	0.0173	0.0004	0.0031	0.0003
TG(16:0/20:0/20:0)	TG	0.1297	0.0032	0.0007	0.0000	0.0138	0.0012
HexCer(9:0;2O/34:1)	HexCer	0.0198	0.0005	0.0093	0.0002	0.0021	0.0002
PC(31:4;O)	PC	0.1071	0.0027	0.0002	0.0000	0.0113	0.0010
HexCer(8:0;2O/32:4)	HexCer	0.0370	0.0009	0.0331	0.0008	0.0038	0.0003
TG(16:0/16:0/26:0)	TG	1.2495	0.0312	0.2400	0.0062	0.1275	0.0110
PE(18:1/22:6)	PE	2.2367	0.0559	3.7862	0.0972	0.2223	0.0191
AHexCer((16:1;2O/16:3;O)	AHexCer	0.3202	0.0080	0.0480	0.0012	0.0312	0.0027
Hex2Cer(40:1;2O)	Hex2Cer	0.9766	0.0244	0.1129	0.0029	0.0923	0.0079
HexCer(16:1;2O/28:1;O)	HexCer	0.0398	0.0010	0.0200	0.0005	0.0036	0.0003
PC(15:0;O/20:4)	PC	0.2779	0.0069	0.0000	0.0000	0.0246	0.0021
TG(18:2/18:2/18:2)	TG	12.8812	0.3220	0.4397	0.0113	1.0791	0.0929
HexCer(18:3;3O/26:2;(2OH))	HexCer	3.7670	0.0942	3.5031	0.0899	0.3125	0.0269
LNAPE(18:2/N/18:2)	LNAPE	1.4245	0.0356	1.4635	0.0376	0.1138	0.0098
CAR(24:1)	CAR	0.1476	0.0037	0.0071	0.0002	0.0109	0.0009
Cer(23:0;2O/44:12)	Cer	0.0185	0.0005	0.0022	0.0001	0.0014	0.0001
Cer(33:0;2O/13:0)	Cer	0.0071	0.0002	0.0015	0.0000	0.0005	0.0000

HexCer(16:1;2O/26:1;O)	HexCer	0.3187	0.0080	0.2059	0.0053	0.0215	0.0018
PE(26:0;O/24:3)	PE	6.5815	0.1645	0.0028	0.0001	0.4229	0.0364
PE(18:2;O/24:1)	PE	0.2341	0.0059	0.1667	0.0043	0.0140	0.0012
SHexCer(14:1;2O/28:2)	SHexCer	0.0863	0.0022	0.1523	0.0039	0.0051	0.0004
SHexCer(14:0;2O/34:1)	SHexCer	2.4299	0.0607	0.0000	0.0000	0.1349	0.0116
HexCer(18:1;2O/24:2)	HexCer	0.6177	0.0154	0.0000	0.0000	0.0342	0.0029
TG(14:0/22:5/22:5)	TG	12.8373	0.3209	0.0000	0.0000	0.6943	0.0597
PC(18:3/18:3)	PC	0.0241	0.0006	0.0105	0.0003	0.0013	0.0001
AHexCer(16:1;2O/14:0;O)	AHexCer	0.0046	0.0001	0.0021	0.0001	0.0002	0.0000
Cer(33:0;2O/15:0)	Cer	0.0032	0.0001	0.0011	0.0000	0.0002	0.0000
TG(9:0/9:0/38:6)	TG	2.0457	0.0511	0.0016	0.0000	0.1051	0.0090
PC(12:0;O/28:6)	PC	0.2960	0.0074	0.0012	0.0000	0.0147	0.0013
PEtOH(24:0/18:1)	PEtOH	0.0782	0.0020	0.0109	0.0003	0.0039	0.0003
TG(18:0/18:1/26:0)	TG	0.1533	0.0038	0.0160	0.0004	0.0075	0.0006
PC(28:5;O/18:1)	PC	5.0858	0.1271	0.0033	0.0001	0.2359	0.0203
PC(43:7)	PC	0.0149	0.0004	0.0008	0.0000	0.0007	0.0001
Cer(28:0;2O/19:0;(2OH))	Cer	0.0003	0.0000	0.0000	0.0000	0.0000	0.0000
DGGA(21:2/26:2)	DGGA	1.3007	0.0325	0.0424	0.0011	0.0526	0.0045
PA(23:0/20:4)	PA	0.0454	0.0011	0.0000	0.0000	0.0018	0.0002
PE(17:1;O/22:5)	PE	0.6492	0.0162	0.2339	0.0060	0.0253	0.0022
HexCer(10:0;2O/44:2)	HexCer	0.0157	0.0004	0.0025	0.0001	0.0006	0.0001
Cer(27:0;2O/19:0;(2OH))	Cer	0.0032	0.0001	0.0009	0.0000	0.0001	0.0000

PC(49:7)	PC	2.0150	0.0504	0.0000	0.0000	0.0744	0.0064
PC(49:10)	PC	0.0003	0.0000	0.0000	0.0000	0.0000	0.0000
CAR(24:3)	CAR	0.3580	0.0090	0.0076	0.0002	0.0118	0.0010
CAR(24:2)	CAR	0.4285	0.0107	0.0222	0.0006	0.0131	0.0011
PI(15:1/22:6)	PI	0.0547	0.0014	0.0180	0.0005	0.0016	0.0001
Cer(14:0;2O/16:0;O(FA 17:0))	Cer	1.1880	0.0297	0.1590	0.0041	0.0352	0.0030
TG(16:0/18:3/22:6)	TG	6.9563	0.1739	0.0008	0.0000	0.2038	0.0175
AHexCer(16:1;2O/14:0;O)	AHexCer	0.0067	0.0002	0.0027	0.0001	0.0002	0.0000
Cer(29:0;2O/15:0)	Cer	0.0205	0.0005	0.0017	0.0000	0.0005	0.0000
HexCer(11:0;2O/44:8)	HexCer	4.7056	0.1176	0.0095	0.0002	0.1148	0.0099
AHexCer(16:1;2O/14:0;O)	AHexCer	0.0514	0.0013	0.0000	0.0000	0.0012	0.0001
PC(28:5/16:0)	PC	2.2709	0.0568	0.0000	0.0000	0.0504	0.0043
TG(16:1/22:0/22:0)	TG	0.4286	0.0107	0.0020	0.0001	0.0095	0.0008
Cer(14:0;2O/19:0;O(FA 16:0))	Cer	0.7459	0.0186	0.1263	0.0032	0.0150	0.0013
Cer(24:0;2O/38:2)	Cer	0.0213	0.0005	0.0108	0.0003	0.0004	0.0000
SMGDG(18:0;O/28:0)	SMGDG	5.9381	0.1485	0.1006	0.0026	0.1133	0.0097
TG(8:0/22:4/28:6)	TG	8.5510	0.2138	0.0000	0.0000	0.1509	0.0130
Cer(14:0;2O/36:0;(2OH))	Cer	0.0006	0.0000	0.0003	0.0000	0.0000	0.0000
DGGA(27:0/22:5)	DGGA	6.4237	0.1606	0.0307	0.0008	0.1065	0.0092
TG(14:0;O/22:0/22:0)	TG	0.2288	0.0057	0.0007	0.0000	0.0033	0.0003
AHexCer(16:1;2O/16:3;O)	AHexCer	3.2260	0.0807	0.0375	0.0010	0.0449	0.0039
Cer(20:0;2O/22:6;(3OH)(FA 18:2))	Cer	0.7624	0.0191	0.0000	0.0000	0.0099	0.0009

SHexCer(14:0;2O/34:2)	SHexCer	3.9198	0.0980	0.0625	0.0016	0.0472	0.0041
Cer(20:0;2O/22:6;(3OH)(FA 18:1))	Cer	0.0771	0.0019	0.0000	0.0000	0.0008	0.0001
Cer(14:0;2O/32:1;(2OH))	Cer	0.0010	0.0000	0.0005	0.0000	0.0000	0.0000
PI(16:0/20:3;O)	PI	0.0010	0.0000	0.0150	0.0004	0.0000	0.0000
AHexCer(16:1;2O/17:3;O)	AHexCer	0.9641	0.0241	0.0000	0.0000	0.0096	0.0008
SHexCer(48:3;3O)	SHexCer	1.2918	0.0323	0.0000	0.0000	0.0116	0.0010
PI(2:0/5:0)	PI	0.0014	0.0000	0.0013	0.0000	0.0000	0.0000
Cer(18:0;2O/22:6;(3OH)(FA 18:1))	Cer	0.2486	0.0062	0.0001	0.0000	0.0015	0.0001
PEtOH(16:0/26:4)	PEtOH	0.0018	0.0000	0.0000	0.0000	0.0000	0.0000
PC(46:5)	PC	0.4605	0.0115	0.0000	0.0000	0.0026	0.0002
Cer(25:0;2O/44:12)	Cer	0.0175	0.0004	0.0030	0.0001	0.0001	0.0000
HexCer(12:0;2O/44:2)	HexCer	0.0175	0.0004	0.0029	0.0001	0.0001	0.0000
TG(18:0;O/20:1/20:1)	TG	2.7280	0.0682	0.0016	0.0000	0.0137	0.0012
PC(43:0)	PC	1.6089	0.0402	0.0000	0.0000	0.0075	0.0006
PG(17:0/20:1)	PG	0.0026	0.0001	0.0000	0.0000	0.0000	0.0000
LNAPE(16:0/N/24:2)	LNAPE	0.0027	0.0001	0.0040	0.0001	0.0000	0.0000
Cer(18:0;3O/26:0;(2OH))	Cer	0.0029	0.0001	0.0000	0.0000	0.0000	0.0000
AHexCer(16:1;2O/14:0;O)	AHexCer	0.0030	0.0001	0.0014	0.0000	0.0000	0.0000
PI(15:0)	PI	0.0036	0.0001	0.0000	0.0000	0.0000	0.0000
PI/Cer(12:0;2O/26:2;O)	Cer	0.0040	0.0001	0.0027	0.0001	0.0000	0.0000
HexCer(16:3;2O/42:0;O)	HexCer	0.0041	0.0001	0.0026	0.0001	0.0000	0.0000
Cer(22:0;2O/40:2;(2OH))	Cer	0.0043	0.0001	0.0015	0.0000	0.0000	0.0000

Cer(22:1;2O/36:1)	Cer	0.0424	0.0011	0.0087	0.0002	0.0001	0.0000
PC(35:0)	PC	0.0051	0.0001	0.0037	0.0001	0.0000	0.0000
PI(35:0)	PI	0.0053	0.0001	0.0000	0.0000	0.0000	0.0000
SMGDG(9:0;O/26:3)	SMGDG	0.0069	0.0002	0.0120	0.0003	0.0000	0.0000
HexCer(14:0;2O/44:2)	HexCer	0.0084	0.0002	0.0011	0.0000	0.0000	0.0000
DG(O/16:1;O/20:4)	DG	0.0099	0.0002	0.0000	0.0000	0.0000	0.0000
Cer(8:0;2O/42:1)	Cer	0.0117	0.0003	0.0010	0.0000	0.0000	0.0000
HexCer(21:0;2O/44:10)	HexCer	0.0130	0.0003	0.0126	0.0003	0.0000	0.0000
Cer(10:0;2O/44:2)	Cer	0.0132	0.0003	0.0011	0.0000	0.0000	0.0000
SMGDG(18:0;O/28:1)	SMGDG	21.0353	0.5259	0.0000	0.0000	0.0153	0.0013
PE(15:1;O/18:1)	PE	0.0153	0.0004	0.0607	0.0016	0.0000	0.0000
PG(18:1/20:3;O)	PG	0.0164	0.0004	0.0185	0.0005	0.0000	0.0000
Cer(8:0;2O/44:1)	Cer	0.0169	0.0004	0.0015	0.0000	0.0000	0.0000
HexCer(9:0;2O/34:2)	HexCer	0.0388	0.0010	0.0168	0.0004	0.0000	0.0000
HexCer(18:1;2O/23:0)	HexCer	0.0462	0.0012	0.0000	0.0000	0.0000	0.0000
PC(16:3;O/24:6)	PC	0.0548	0.0014	0.0000	0.0000	0.0000	0.0000
Hex2Cer(29:2;2O)	Hex2Cer	0.0585	0.0015	0.0281	0.0007	0.0000	0.0000
PC(36:1)	PC	0.0624	0.0016	0.0096	0.0002	0.0000	0.0000
AHexCer(16:1;2O/14:1;O)	AHexCer	0.0647	0.0016	0.0019	0.0000	0.0000	0.0000
PC(38:1)	PC	0.1164	0.0029	0.0215	0.0006	0.0000	0.0000
Cer(15:0;2O/44:11)	Cer	0.1693	0.0042	0.1116	0.0029	0.0000	0.0000
HexCer(17:0;2O/23:1;O)	HexCer	0.2428	0.0061	0.1144	0.0029	0.0000	0.0000

PE(19:1;O/18:1)	PE	0.2869	0.0072	0.1536	0.0039	0.0000	0.0000
HexCer(16:1;2O/25:0;O)	HexCer	0.2923	0.0073	0.0995	0.0026	0.0000	0.0000
PS(14:1/24:4)	PS	0.4527	0.0113	0.2444	0.0063	0.0000	0.0000
TG(18:2/18:2/18:3)	TG	0.5261	0.0132	0.0000	0.0000	0.0000	0.0000
Cer(14:0;2O/28:2;(2OH))	Cer	0.6121	0.0153	0.2944	0.0076	0.0000	0.0000
HexCer(18:1;2O/24:1)	HexCer	0.9768	0.0244	0.4057	0.0104	0.0000	0.0000
PE(18:2;O/22:1)	PE	1.4302	0.0358	1.3943	0.0358	0.0000	0.0000
Cer(14:0;2O/26:1;O(FA 17:0))	Cer	2.0417	0.0510	0.3245	0.0083	0.0000	0.0000
TG(18:2/18:3/18:3)	TG	2.2545	0.0564	0.0000	0.0000	0.0000	0.0000
HexCer(12:2;2O/44:12)	HexCer	3.4397	0.0860	0.0000	0.0000	0.0000	0.0000
PE/Cer(16:1;2O/26:2;O)	Cer	6.7503	0.1688	6.3634	0.1634	0.0000	0.0000
